# Multi-task adversarial autoencoder for functional genomic element generation with preserved biophysical properties

**DOI:** 10.1186/s13040-026-00591-9

**Published:** 2026-08-03

**Authors:** Shamsuddeen Adamu, Hitham Alhussian, Said Jadid Abdulkadir, Majdy Mohamed Eltayeb Eltahir, Sallam O. F. Khairy, Ibrahim Hayatu Hassan, Ibrahim Muhammad Kurah, Abubakar Mukhtar, Vijayakanthan Ganesalingam, Vaishali Ravi, Yahaya Saidu

**Affiliations:** 1 Computer Information Science Department, Universiti Teknologi PETRONAS, Seri Iskandar, Malaysia; 2 IAIICT, Ahmadu Bello University, Zaria, Nigeria; 3 King Khalid University, Applied College of Muhayil Asir, Abha, Saudi Arabia; 4 Department of Information Systems, College of Economics, Management and Information Systems, University of Nizwa, Al Khuwair, Oman

**Keywords:** Generative adversarial networks, Adversarial autoencoders, Multi-task learning, Genomic sequence generation, Genomic data augmentation

## Abstract

Generative modeling of genomic sequences presents a stringent test for deep learning, requiring the capture of long-range dependencies and functional constraints beyond local nucleotide statistics. Existing architectures frequently collapse to limited modes or reproduce shallow nucleotide distributions without encoding functional semantics. We introduce the Multi-Task Adversarial Autoencoder (MT-AAE), a hybrid generative framework that integrates adversarial regularization with auxiliary functional and biophysical objectives to enforce structured latent representations. Evaluated on an empirical human gene corpus, MT-AAE achieved a Train-on-Synthetic-Test-on-Real (TRTS) accuracy of 74.7%, compared with 41.0% for a standard GAN baseline. Stratified analysis further showed that functional discriminability increased to 89.3% when sequence lengths aligned with the model’s architectural window. Importantly, the learned representations exhibited emergent biological structure: synthetic sequences spontaneously preserved *cis*-regulatory syntax, including canonical TATA-box motifs recovered across 100% of generated promoter sequences without explicit rule encoding, though positional placement relative to the TSS was not statistically significant (KS *p=0.90*), and the high occurrence rate is partly attributable to the AT-rich composition of the generated sequences. Representation-level validation using frozen DNABERT-2 and DNABERT-S embeddings confirmed that the generated sequences retained functional information beyond shallow k-mer statistics. Cross-species evaluation on *Mus musculus* sequences further demonstrated species-specific learning consistent with known human–mouse regulatory divergence. The framework also mitigated mode collapse, maintaining near-uniform generation across functional classes ($R_g \approx 1.0$), including rare categories such as tRNAs (* < 2%* of the dataset). These findings position MT-AAE as an effective framework for biologically constrained genomic sequence generation.

**Supplementary Information:**

The online version contains supplementary material available at https://doi.org/10.1186/s13040-026-00591-9.

## Introduction

Computational genomics enables the design of synthetic DNA sequences, such as promoters and enhancers, to advance synthetic biology and therapeutic applications [1, 2]. These sequences must exhibit precise functional properties, including transcription factor binding and gene expression regulation, while maintaining biophysical characteristics like GC-content, k-mer frequencies, and chromatin accessibility. The ability to generate such sequences computationally supports applications ranging from gene therapy and mRNA vaccine design to metabolic engineering, where tailored genomic elements drive targeted biological outcomes [3, 4].

Generating synthetic genomic elements remains challenging due to the complexity of preserving both functional and biophysical properties [5]. This is a long-standing challenge in synthetic biology, as traditional methods are often time-consuming, inefficient, yielding outcomes often insufficient for reliable application [6]. Despite the advent of deep generative models, these limitations persist. In vivo validation reveals that a significant portion, up to 40% of synthetic promoters still fail to achieve expected regulatory effects, often due to deviations in sequence composition or structural constraints [7]. These failures incur significant costs in time and resources, limiting the scalability of synthetic biology applications. The need for sequences that generalize across diverse cell types and maintain empirical statistical distributions highlights the urgency of addressing these challenges [6].

Existing generative models, such as Variational autoencoder (VAEs) and Generative adversarial network (GANs), excel at capturing local motifs but suffer from critical limitations. VAEs often face posterior collapse [8, 9], reducing sample fidelity, while GANs struggle with mode collapse in underrepresented genomic classes [10]. More recent architectures, such as Transformer-based models, effectively handle long-range dependencies but are typically hindered by fixed-length constraints that are ill-suited to variable-length genomic elements like promoters [11, 12]. Multi-task learning (MTL) frameworks, such as those integrating chromatin accessibility and gene expression prediction, improve generalization across predictive tasks but rarely extend to de novo multi-omic generation or conditional synthesis [13]. Crucially, existing frameworks rarely incorporate multi-objective optimization to simultaneously balance functional accuracy with multiple biophysical constraints, nor do they provide interpretable latent representations for biological validation [14, 15].

This work addresses three primary research gaps: the inability of existing models to handle variable-length elements, the absence of frameworks that jointly integrate multiple biophysical constraints, and the limited interpretability of generated sequences. To resolve these challenges, we developed a Multi-Task Adversarial Autoencoder (MT-AAE) capable of generating variable-length genomic elements with high functional and biophysical fidelity. The model applies an adversarial training regime to encourage realism and incorporates auxiliary tasks to regularize the latent space, which strengthens biophysical preservation and enhances predictive utility for downstream biological analyses.

The key contributions of this study are:

- A novel Multi-Task Adversarial Autoencoder (MT-AAE) architecture that fuses adversarial training with biophysical auxiliary losses to alleviate mode collapse and posterior divergence in genomic generation.
- A context-aware length normalization strategy that allows a fixed-length generative model to accommodate variable-length genomic inputs while preserving functional content and avoiding artifacts associated with naive padding or truncation.
- An interpretable latent space representation designed to enhance trust in synthetic sequences for therapeutic use.
- A comprehensive evaluation protocol incorporating Train-on-Synthetic, Test-on-Real (TRTS) utility metrics and Jensen–Shannon divergence (JSD) to validate both functional and biophysical fidelity [16, 17].

The MT-AAE framework addresses critical limitations in synthetic biology and facilitates more reliable data augmentation for rare genomic classes. This capability stands to accelerate genomic research, as high-quality synthetic data can mitigate privacy concerns in large-scale biobanks and facilitate the design of bespoke therapeutic constructs [12].

The rest of this paper is organized as follows: section “Literature review” reviews the literature on deep learning, generative models, multi-task learning, and evaluation metrics in computational genomics. Section “Methodology” presents the proposed MT-AAE methodology. Section “Results” details the experimental setup and evaluation. Section “Discussion” discusses results and implications, and Section 6 concludes with future directions.

## Literature review

### Deep learning in genomic sequence analysis

Deep learning has reshaped computational genomics by enabling models to learn hierarchical sequence-to-function mappings directly from raw DNA [1, 2]. Early architectures were dominated by convolutional neural networks (CNNs), which effectively identified regulatory motifs and cis-elements through localized pattern detection, analogous to image filters [6, 18]. Although CNNs achieved high accuracy in transcription factor binding site prediction, they struggled to capture distal dependencies along genomic sequences. This limitation led to the adoption of recurrent neural networks (RNNs), with Long Short-Term Memory (LSTM) variants emerging as the leading approach for modeling sequential relationships in variable-length inputs, enabling advances in applications such as splice site and RNA structure prediction [19].

The current paradigm has shifted toward transformer architectures, which employ self-attention to model long-range dependencies without the vanishing-gradient limitations of RNNs [11, 20]. Attention mechanisms have proven central to motif discovery, allowing models such as DNABERT to outperform conventional position weight matrices by learning cooperative interactions among distant genomic regions [21]. Hybrid attention–CNN architectures further refined this capability, achieving state-of-the-art accuracy in de novo motif discovery across diverse cell types [22].

Many of these advances are structured around encoder–decoder frameworks, adapted from sequence-to-sequence (seq2seq) models originally developed for machine translation [23]. These frameworks are well-suited to genomics, bridging biological modalities such as translating genomic coordinates to functional annotations or predicting epistatic interactions [24, 25]. This encoder–decoder foundation, which maps sequences into latent representations and reconstructs functional outputs, established the architectural basis for the next frontier: de novo generative modeling.

### Generative models in computational biology

Generative modeling in computational biology has evolved from classical probabilistic formulations to deep neural architectures capable of capturing the high-dimensional structure of genomic sequence space [26]. VAEs have become a foundational approach for sequence generation, using latent variable models to encode complex regulatory patterns into compact, continuous representations that capture major sources of biological variation [27]. For example, BindVAE incorporates Dirichlet priors to promote motif-aware structure in the latent space, enabling the synthesis of enhancer elements that retain cell-type–specific sequence features [28]. This capacity for probabilistic latent-space interpolation proves especially advantageous in low-data scenarios, where they can enrich rare genomic subclasses or underrepresented functional states without requiring explicit motif engineering.

GANs provide a complementary paradigm, using adversarial training to encourage the production of high-fidelity, sharply defined sequences reflective of empirical regulatory landscapes [29]. GAN variants have been applied to promoter design, regulatory grammar exploration, and de novo construction of biologically active sequences [30]. Feedback-regulated GANs (FBGANs) further integrate functional or biophysical scoring signals during training, guiding the generator toward sequences that satisfy multiple biological constraints and enabling practical generation of sequences across diverse lengths [31]. Outside of classical regulatory genomics, GAN-based frameworks have also been used to engineer synthetic genomes optimized for DNA data storage, including error-resilient sequence mimics of large mammalian genomes [32]. Recent work has substantially expanded the toolkit available for generative genomic modelling. DNADiffusion [33] applies denoising diffusion probabilistic models to the design of cell-type-specific regulatory sequences, reporting high motif fidelity and chromatin accessibility in the generated elements. Evo [34], a Hyena-based autoregressive model trained at genome scale, achieves strong performance in long-range sequence generation and variant-effect prediction. Geneformer [35] fine-tunes transformer representations for gene regulatory network inference, operating primarily as a discriminative framework rather than a generative model. These approaches represent the current frontier of large-scale genomic sequence learning. Their primary design objectives differ from those of MT-AAE, however, as none jointly enforces biophysical constraints, class-conditional generation across imbalanced functional categories, and interpretable latent structure within a compact framework suitable for data augmentation under resource constraints.

Despite these progress, generative frameworks for de novo functional genomic element design face core limitations. Many models impose fixed-length outputs, ill-suited to the intrinsic variability of regulatory elements. Even when length-flexible, their outputs often diverge from empirical distributions in critical higher-order statistics—such as k-mer composition and GC-content—which govern molecular stability and protein-DNA interactions [36, 37]. Architecturally, GANs remain prone to mode collapse [10], eroding diversity for rare classes, while VAEs frequently suffer from posterior collapse [8], leading to an impoverished latent space that fails to preserve essential regulatory structure.

Although conditional architectures, reinforcement-guided optimization, and physics-informed constraints have improved specific facets of generative fidelity [38], a fundamental gap persists. Current methodologies lack the capacity to jointly optimize the multi-objective requirements of biological sequence design—functional discriminability, motif integrity, global statistical coherence, and biophysical plausibility. Instead, they often prioritize a subset of these criteria, failing to produce fully functional and biomimetically consistent sequences.

The importance of biophysical coherence in generative genomics is further underscored by recent work on chromatin organization and epigenome-transcriptome coupling. Sefer [39] demonstrated that Hi-C interaction graphs can be decomposed in terms of histone modification pairs, identifying four histone marks (H3K4me1, H3K4me3, H3K9me3, H3K27ac) as broadly predictive of chromatin interaction patterns across species and cell types. This analysis directly motivates the inclusion of biophysical constraints in generative models, since synthetic sequences that deviate from these higher-order chromatin signatures are unlikely to be functional in cellular contexts. Complementarily, Sefer [40] proposed ProbC, a probabilistic framework for jointly modeling epigenomic and transcriptomic effects in three-dimensional genome organization. ProbC highlights that functional genomic element generation cannot be evaluated solely at the sequence level but must account for the broader regulatory landscape encoded in chromatin structure. These works collectively reinforce the rationale of the MT-AAE’s multi-objective design: while prior generative models have focused primarily on sequence statistics, robust generation of biologically active elements requires alignment with the epigenomic and structural constraints that govern transcriptional competence in vivo.

Closing this gap is paramount for advancing trustworthy synthetic genomics and for constructing biologically meaningful data-augmentation pipelines. Table 1 systematically contrasts these limitations across prevailing frameworks, highlighting the need for an integrated approach.

**Table 1:** Comparative analysis of genomic sequence generation frameworks

Framework	Architecture	Class Balance	Multi-Constraint Optimization	Interpretability	Key Limitations
BindVAE [28]	VAE + Dirichlet priors	No	Motif preservation only	Partial (motif-aware)	Fixed-length, posterior collapse
FBGAN [31]	GAN + feedback	Limited	Functional scoring only	Limited	Mode collapse in rare classes
DNABERT [21]	Transformer	No	Single-task focus	Attention maps only	Fixed-length constraints
UnitedNet [41]	MTL predictor	Non-generative	Multi-omic prediction	Cross-omic relationships	Not generative
**MT-AAE**	**MTL + Adversarial AE**	**Aggressive sampling**	**Functional + biophysical**	**Structured latent space**	**Fixed-length processing**

These architectural limitations become most apparent in functional genomic element design, a setting that requires the simultaneous preservation of motif structure, global sequence statistics, and biophysical properties to maintain biological activity.

### Multi-task learning in bioinformatics

MTL enhances generalization in bioinformatics by enabling models to share representations across biologically related objectives [42]. In genomics, MTL is applied to concurrent prediction of chromatin accessibility, transcription factor binding, and methylation states, capturing the interdependencies that govern regulatory function [43]. Joint optimization of complementary tasks such as variant-effect prediction and motif scoring—supports transfer learning while reducing reliance on extensive task-specific fine-tuning [44].

Transfer learning across sequence-derived properties, including gene expression, chromatin states, and local epigenetic contexts, mitigates overfitting in sparsely annotated genomic datasets [45]. Beyond data efficiency, MTL introduces intrinsic regularization: auxiliary tasks act as inductive biases that reinforce biophysical consistency and stabilize training dynamics [46]. For example, UnitedNet integrates single-cell multi-omics data to jointly infer gene expression and chromatin accessibility, revealing interpretable cross-omic relationships [41]. Bayesian extensions of this paradigm advance precision medicine by co-modeling drug responses with patient-specific genomic profiles [47].

Despite these established benefits, current generative frameworks seldom employ MTL as an architectural mechanism for unifying functional, compositional, and structural constraints. This gap limits their ability to generate sequences that maintain biological coherence across multiple regulatory dimensions. The proposed MT-AAE addresses this limitation by embedding multi-task supervision directly into the generative process, thereby improving both sequence fidelity and regulatory plausibility. Evaluating such multi-constraint generative models requires integrated metrics capable of jointly assessing functional accuracy, statistical coherence, and structural validity.

### Evaluation metrics for synthetic biological data

Evaluating synthetic genomic sequences requires multi-faceted assessment that extends beyond standard generative losses. The TRTS framework establishes biological utility by measuring downstream predictive performance on empirical datasets [48]. This functional evaluation reveals limitations often obscured by likelihood-based metrics, especially in regulatory element prediction contexts where synthetic sequences must retain discriminative capacity on authentic biological benchmarks [5].

Distributional fidelity is assessed using statistical divergence measures such as Jensen-Shannon divergence (JSD), Kolmogorov-Smirnov (KS) tests, and k-mer frequency analyses across multiple sequence resolutions [49, 50]. When interpreted through mutual-information-based symmetrization, JSD captures deviations in GC-content, dinucleotide composition, and related higher-order features that influence nucleosome positioning and DNA mechanics [51]. KS testing complements this approach by evaluating cumulative distribution agreement for biophysical properties, including melting temperature and structural flexibility profiles [52].

Preservation of regulatory grammar is examined through motif-centered tools such as the MEME Suite and AIModules, which quantify transcription factor binding site enrichment, positional conservation, and motif co-occurrence patterns [53]. These frameworks facilitate comparison of motif logos between synthetic and empirical sequences, revealing distortions in cooperative binding architectures and combinatorial regulatory logic [54]. Despite their utility, existing evaluation pipelines face scalability challenges for ultra-long sequences and lack integration with privacy-preserving metrics such as differential privacy epsilon bounds [48], which limits their application to genome-scale synthetic data generation.

### Research gaps and unaddressed challenges

Substantial advances in generative modeling have not eliminated core limitations that restrict the reliable design of functional genomic sequences. Variable-length generation remains a central challenge. Most architectures rely on fixed-length windows, which constrain representation of regulatory elements whose boundaries are inherently heterogeneous, including enhancers, introns, and operons [11, 55]. This constraint is further amplified by unresolved assembly gaps in reference genomes, which introduce structural uncertainties that propagate into learned sequence distributions during training [56].

A second unresolved issue concerns the balance between functional and biophysical fidelity. Models often excel in capturing motif-level patterns while failing to reproduce broader compositional and structural properties such as thermodynamic stability, secondary structure profiles, and long-range k-mer statistics [26]. These fidelity gaps are compounded by limitations in evaluation scalability. TRTS assessment becomes infeasible for genome-scale sequences because memory consumption grows quadratically with sequence length, preventing comprehensive benchmarking across full genomic contexts [48].

Interpretability remains underdeveloped in generative sequence frameworks. Current models provide limited visibility into how latent variables encode regulatory dependencies or biophysical constraints, which restricts their applicability in settings that demand mechanistic insight or rigorous safety assessment [57]. Addressing these limitations requires generative systems that integrate multi-task supervision, regulatory constraint modeling, and transparent latent structure. The framework introduced in this study advances this direction by unifying functional prediction, structural coherence, and interpretable sequence generation within a single architecture that directly addresses these interconnected challenges.

## Methodology

### Dual-phase dataset framework

To systematically evaluate the MT-AAE architecture under both controlled and realistic genomic conditions, we adopted a dual-phase dataset framework. This approach enabled mechanistic validation in tightly constrained synthetic environments while assessing empirical performance on natural genomic variation.

#### Phase I: Synthetic benchmark for mechanistic validation

Phase I established a controlled environment for examining the model’s capacity to recognize regulatory grammar without interference from evolutionary drift or stochastic mutational processes. We generated a rule-based synthetic corpus ($\mathcal{D}_{syn}$) containing 1,850 sequences through stochastic sampling of predefined biological motifs. Each sequence class incorporated specific biophysical rules, conserved regulatory elements, and deterministic motif placements to form precise ground truth for mechanistic analysis. This configuration enabled direct evaluation of whether the model captures functional signatures embedded in regulatory architecture rather than superficial sequence regularities. Table 2 outlines the dataset’s composition, length distributions, GC characteristics, and the biological constraints applied during construction.

**Table 2:** Composition and design rules of the synthetic benchmark dataset ($\mathcal{D}_{syn}$)

Gene Class	N	Length (bp)	GC *μ*	Embedded Biological Constraints
Protein Coding	300	150–500	0.45	Canonical ORFs (Start: ATG, Stop: TAA/TAG/TGA)
Promoter	200	100–200	0.60	Assembly platforms (TATA-box: TATAAA, CAAT-box)
Intron	250	80–300	0.40	Splicing motifs (Donor: GTAAGT, Branch: CTGAC)
Enhancer	150	100–250	0.50	TF binding sites (GATA, CAAT), distal regulation
ncRNA	200	70–150	0.55	Structural scaffolds (Poly-G/C), non-coding preservation
snoRNA	30	60–150	0.52	Nucleolar localization signals (TGATGA)
tRNA	20	70–90	0.54	Anticodon stems (GTCG), cloverleaf architecture
**Total**	**1,850**	**60–500**	**0.40–0.60**	**Deterministic motif injection**

#### Phase II: NCBI human gene corpus for empirical evaluation

Phase II broadened the evaluation to large-scale empirical genomic data to assess performance under realistic biological heterogeneity. We obtained 27,346 human gene sequences from the NCBI Gene Database [58] ($\mathcal{D}_{emp}$) as the raw source corpus. Following preprocessing—including length filtering (retaining sequences of 60–5,000 bp), removal of sequences with ambiguous nucleotide content exceeding 5%, and class-label assignment—1,910 sequences were retained for model training and evaluation. The processed dataset was partitioned into training (60.0%), validation (20.0%), and held-out test (20.0%) sets, as summarized in Table 3. This stratification supported model optimization, hyperparameter calibration, and unbiased evaluation under the TRTS protocol.

**Table 3:** Partitioning and statistics of the NCBI empirical human gene corpus ($\mathcal{D}_{emp}$)

Partition	Sequence Count	Distribution (%)	Role
Training Set	1,146	60.0%	Model Optimization
Validation Set	382	20.0%	Hyperparameter Tuning
Test Set	382	20.0%	Unseen Evaluation (TRTS)
**Total**	**1,910**	**100.0%**	**–**

Properties: Human gene sequences (60–5,000 bp), Nucleotide Alphabet $\Sigma = \left\{ {A,T,C,G} \right\}$

To confirm that the random partition does not introduce homology-based leakage between training and test sets, all 1,910 processed sequences were clustered at 80% nucleotide identity using CD-HIT-EST [59]. The clustering produced 1,910 groups, none of which contained sequences from both partitions simultaneously, yielding a contamination rate of 0% (0 of 382 test sequences). The train/test boundary is therefore free of sequence-level homology overlap, and the reported TRTS performance reflects generalisation to genuinely unseen sequences rather than near-duplicate retrieval.

#### Class imbalance correction strategy

Addressing the severe class imbalance inherent in genomic datasets, where regulatory elements such as snoRNAs and tRNAs are naturally underrepresented, required a dual-strategy approach [60]. We combined strategic oversampling with weighted loss functions to ensure adequate representation of minority classes. The stratified balanced sampling algorithm (Algorithm 1) enforced minimum representation thresholds through strategic oversampling while maintaining proportional sampling for majority classes. This approach worked in tandem with class-weighted loss functions and a custom monitoring callback that provided real-time feedback on minority class performance, enabling dynamic curriculum adaptation during training.

**Figure:**
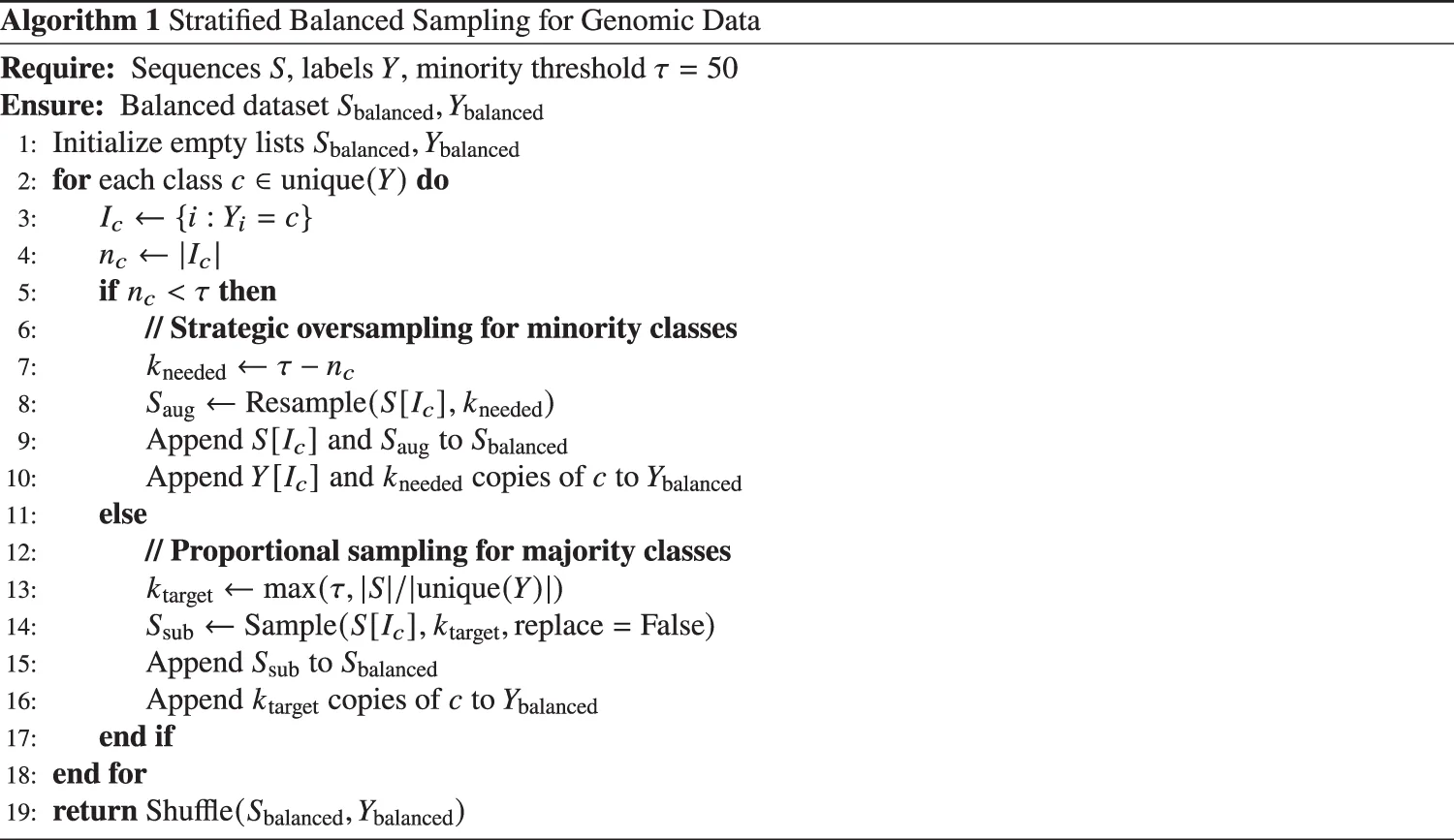

### Sequence representation and mathematical formalization

Methodological consistency mandated identical preprocessing across both experimental phases to ensure comparable evaluations. Each biological sequence $s \in \mathcal{S}$ was mapped to a discrete tensor representation $\phi: \mathcal{S} \to \{0,1\}^{L \times 4}$, where the fixed length *L = 500* captured the functional domain span while remaining computationally tractable.

#### Length normalization and one-hot encoding

Dimensional homogeneity was strictly enforced through adaptive length normalization to maintain consistent input dimensions across all sequences. Sequences with *|s| < L* underwent central zero-padding, while sequences exceeding *L* were truncated to preserve functional cores. This standardization mitigated vanishing gradient artifacts typical in RNN-based architectures and ensured computational efficiency during batch processing [61].

#### Ambiguous nucleotide imputation

Non-canonical nucleotides (e.g., ‘N’) were resolved via context-aware imputation to preserve local sequence characteristics. We sampled replacement nucleotides from the local empirical distribution $\{A, T, C, G\}$ observed within a flanking 10-base pair window. This approach maintained local GC content and Watson-Crick constraints essential for subsequent adversarial learning and biophysical property preservation.

### MT-AAE architecture overview

**Fig. 1:**
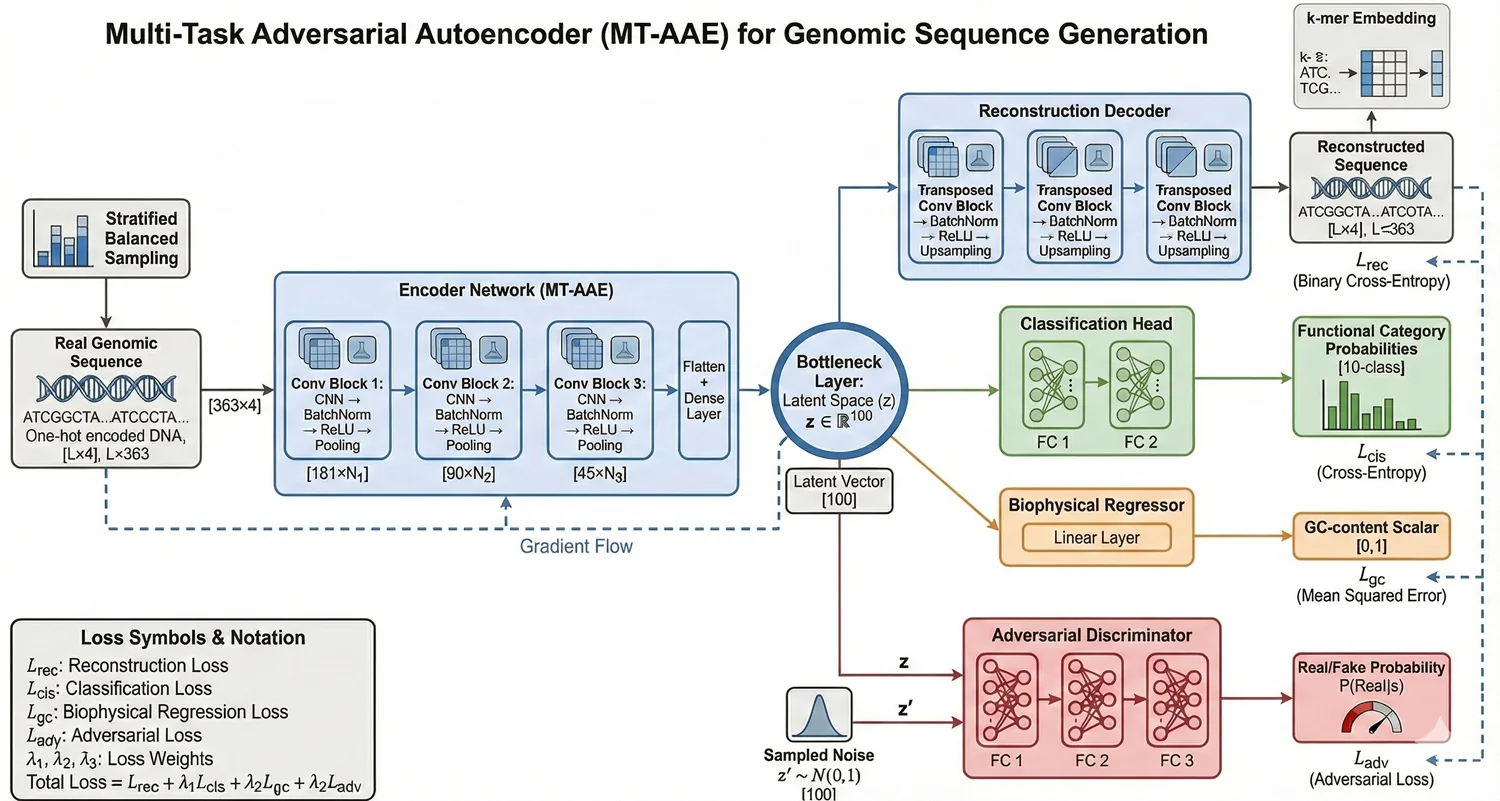
Architecture of the MT-AAE for genomic sequence generation

The MT-AAE framework jointly optimizes four interconnected learning objectives within a single computational graph, connecting encoder *E*, generator *G*, and task-specific heads to enforce both sequence fidelity and biophysical property preservation. As illustrated in Fig. 1, the architecture enforces a latent space that generates sequences statistically indistinguishable from natural DNA while retaining critical functional annotations and structural attributes.

The MT-AAE integrates adversarial training with multi-task learning to generate synthetic DNA sequences that preserve functional and biophysical properties. Key architectural specifications are detailed in Table 4.

**Table 4:** MT-AAE architecture specifications

Component	Specification	Ref.
Sequence Length (*L*)^†^	500	Section “Length normalization and one-hot encoding”
Encoder Type	Bidirectional LSTM (2 layers)	section “Encoder: Bidirectional LSTM with multi-head attention”
Hidden Units	128 per direction	Eq. 1
Attention Mechanism	Multi-Head (8 heads, $d_k=32$)	Eq. 1
Latent Dimension	128	Section “Latent space design and regularization”
Decoder Type	Bidirectional LSTM (2 layers)	Section “Decoder with discrete-DNA output layer”
Output Layer	ValidDNAOutput (Softmax)	Eq. 1
Multi-Task Heads	Class. + GC Reg. + Length	Section “Multi-task prediction heads”
Loss Weights	$\lambda_{rec}=0.3, \lambda_{class}=0.6, \dots$	Eq. 3
Optimizer	Adam (lr = 0.001)	Section “Training dynamics”

†*Note*: *L* = 500 denotes the maximum architectural input dimension including padding

### Core architectural components

#### Encoder: bidirectional LSTM with multi-head attention

The encoder network $E: \mathcal{X} \to \mathcal{Z}$ projected input sequences into a latent manifold using a bidirectional LSTM architecture, explicitly modeling forward and reverse complement dependencies critical for capturing palindromic motifs and bidirectional regulatory elements. For each position *t ∈ [1, L]*, the temporal dynamics captured both sequence directionalities, resulting in a comprehensive representation of genomic regulatory grammar.

The integration of multi-head self-attention mechanisms (*h=8* attention heads with key dimension $d_k = 32$) enabled the model to resolve long-range dependencies and learn position-specific importance weights. This attention mechanism provided the foundation for motif discovery by learning weight distributions that highlight functionally significant positions, with the biological interpretation detailed in section “Motif preservation via attention mechanisms”.

The complete sequence representation was obtained via global average pooling followed by a dense transformation:

1 $$z = W_p \cdot \left( \frac{1}{L} \sum_{t=1}^L h_t \right) + b_p, \quad W_p \in \mathbb{R}^{128 \times 512}, b_p \in \mathbb{R}^{128}$$

where $h_t$ represented the concatenated forward and backward hidden states at position *t*. $L_2$ regularization (*λ = 0.0005*) constrained the weights to prevent overfitting while preserving representational capacity.

#### Latent space design and regularization

The latent space $\mathcal{Z} = \mathbb{R}^{128}$ was dimensioned to balance representational capacity with regularization requirements. We evaluated latent dimensions $\{32, 64, 128, 256\}$ through ablation studies, with 128 providing optimal trade-off between expressiveness for complex genomic patterns and training stability.

Adversarial training regularized this space by minimizing the JSD between the aggregated posterior *q(z)* and an isotropic Gaussian prior $p(z) \sim \mathcal{N}(0, I)$. This enforcement ensured the latent manifold remained continuous, dense, and maximally informative. As a result, semantically meaningful interpolations between functional classes were supported, and pathological discontinuities that typically yield non-functional sequences were eliminated.

#### Decoder with discrete-DNA output layer

The decoder $G: \mathcal{Z} \times \mathcal{Y} \to \mathcal{X}$ reconstructed sequences through a complementary bidirectional LSTM that fused latent representations with class conditioning vectors. The ValidDNAOutput layer constituted a critical architectural constraint guaranteeing every position in the generated sequence represented a valid probability distribution over $\{A, T, C, G\}$.

The decoder output at each position *t* was computed as:

2 $$u_t = W_d h_t^d + b_d \in \mathbb{R}^4, \quad \hat{x}_t = \frac{\exp(u_{t,j})}{\sum_{k=1}^4 \exp(u_{t,k})} \quad \text{for } j \in \{1,2,3,4\}$$

where $h_t^d$ represented the decoder’s hidden state at position *t*.

During training, the differentiable softmax enabled gradient propagation while strictly satisfying the constraint $\sum_{j=1}^4 \hat{x}_{t,j} = 1$ for all positions *t*, ensuring thermodynamic consistency. During inference, sampling from these categorical distributions via argmax yielded discrete nucleotide sequences, preventing physically impossible fractional nucleotide states that could arise in continuous-output models.

#### Multi-task prediction heads

Sequence reconstruction was augmented by three auxiliary tasks designed to enforce preservation of functional annotations and structural properties essential for biological activity. The classification pathway processed pooled sequence representations through a deep network with comprehensive regularization, implementing a dimensional progression of 512 *→* 256 *→* 128 *→* C, where C denotes the sequence class count.

Concurrently, the GC content regression head predicted a fundamental biophysical property encoded in sequence composition, explicitly linking sequence generation to nucleosome positioning preferences. Additionally, biologically plausible sequence length distributions were implicitly learned through the reconstruction objective and explicit padding mechanisms, thereby preserving the characteristic length profiles of their respective functional classes.

### Biophysical property preservation module

#### GC content regression for thermodynamic consistency

The GC regression head enforced explicit preservation of thermodynamic characteristics critical for DNA function. GC content directly influences DNA flexibility, melting temperature ($T_m$), and chromatin accessibility; these structural determinants govern transcriptional competence and regulatory function [62]. By regressing this fundamental biophysical property, the model ensured generated sequences maintained appropriate thermodynamic profiles for biological plausibility.

#### Motif preservation via attention mechanisms

The multi-head attention mechanism enabled explicit identification and preservation of biologically significant regulatory motifs. By learning attention weight distributions that highlight specific sequence positions, the model captured functional elements such as transcription factor binding sites (e.g., TATA-box, CAAT-box), splice donor/acceptor sequences, and promoter elements. This capability ensured these critical regulatory regions were maintained in generated sequences, preserving functional signatures essential for biological activity.

#### Global sequence statistics alignment

Beyond motif-level preservation, the model aligned global sequence statistics to maintain evolutionary constraints encoded in nucleotide composition patterns. We enforced consistency in dinucleotide frequencies, sequence entropy, and length distributions, ensuring generated sequences exhibited statistical properties consistent with natural genomic elements. This comprehensive alignment maintained the complex interdependencies between sequence composition, structure, and function.

### Joint optimization framework and training protocol

#### Multi-task loss formulation

The global optimization objective integrated four loss components with weights determined through extensive empirical validation:

3 $$\mathcal{L}_{\mathrm{total}} = \lambda_{\mathrm{rec}} \mathcal{L}_{\mathrm{rec}} + \lambda_{\mathrm{class}} \mathcal{L}_{\mathrm{class}} + \lambda_{\mathrm{GC}} \mathcal{L}_{\mathrm{GC}} + \lambda_{\mathrm{adv}} \mathcal{L}_{\mathrm{adv}}$$

where $$\lambda_{\mathrm{rec}} = 0.3$$, $$\lambda_{\mathrm{class}} = 0.6$$, $$\lambda_{\mathrm{GC}} = 0.1$$, $$\lambda_{\mathrm{adv}} = 0.001$$.

The reconstruction loss employed categorical cross-entropy across sequence positions:

4 $$\mathcal{L}_{\mathrm{rec}} = -\frac{1}{L} \sum_{t=1}^L \sum_{j=1}^4 x_{t,j} \log(\hat{x}_{t,j})$$

The classification loss incorporated class-weighted categorical cross-entropy to address dataset imbalance:

5 $$\mathcal{L}_{\mathrm{class}} = -\frac{1}{N} \sum_{i=1}^N w_{y_i} \sum_{c=1}^C y_{i,c} \log(\hat{y}_{i,c}), \quad w_{y_i} = \frac{N}{C \cdot |\{j: y_j = y_i\}|}$$

providing increased gradient signal for underrepresented classes.

The GC regression loss utilized mean squared error:

6 $$\mathcal{L}_{\mathrm{GC}} = \frac{1}{N} \sum_{i=1}^N (y_{\mathrm{GC},i} - \hat{y}_{\mathrm{GC},i})^2$$

Finally, the adversarial loss followed the standard GAN objective for latent space regularization.

#### Adversarial latent space regularization

The discriminator $D_z: \mathcal{Z} \to [0,1]$ optimized the minimax objective:

7 $$\min_{E} \max_{D_z} \mathbb{E}_{z \sim p(z)}[\log D_z(z)] + \mathbb{E}_{X \sim p_{\mathrm{data}}}[\log(1 - D_z(E(X)))]$$

This regularization ensured the latent manifold remained continuous and dense, eliminating pathological discontinuities and enabling semantically meaningful interpolations between functional classes.

#### Training dynamics

Training followed an alternating optimization scheme that synchronized updates of the generator components (*E, G*) and latent discriminator ($D_z$) within the multi-task framework (Algorithm 2). Optimization used Adam with a learning rate of *0.001* ($\beta_1=0.9$, $\beta_2=0.999$) and gradient clipping (maximum norm *1.0*) to ensure numerical stability.

Learning rate reduction on plateau (decay factor *0.5*, patience *15* epochs) was combined with checkpointing based on minimum composite validation loss. This approach mitigated overfitting without compromising convergence efficiency.

### Ablation study and efficiency measurement

#### Ablation study design

A five-stage ablation study was conducted to quantify individual component contributions and assess potential task interference within the MT-AAE architecture. Modules were integrated incrementally:

1. **Base AAE**: Reconstruction-only autoencoder establishing baseline generative capacity.
2. **+ Classification**: Addition of a functional classification pathway enforcing class-specific pattern learning.
3. **+ GC Regression**: Inclusion of a GC-content regression head imposing compositional and biophysical constraints.
4. **+ Balanced Sampling**: Strategic oversampling to mitigate class imbalance and improve minority-class representation.
5. **Full MT-AAE**: Complete multi-task model integrating all objectives.

Performance was evaluated at each stage using functional and sequence-quality metrics to characterize both cumulative and synergistic effects of multi-task learning on functional retention, GC-profile alignment, and minority-class recovery.

#### Efficiency measurement

Computational efficiency was systematically quantified to assess practical scalability and resource requirements for large-scale genomic applications. We measured total training time, sequence generation throughput (sequences/second), and memory footprint across three complementary phases: training convergence, sequence generation, and end-to-end pipeline execution. These measurements ensured the MT-AAE framework maintained practical applicability for tasks including synthetic regulatory element design, data augmentation for underrepresented functional classes, and high-throughput in silico mutagenesis studies.

### Comprehensive evaluation pipeline

#### Functional discriminability metrics (TRTS/RTSR)

The TRTS paradigm measured the extent to which functional signals embedded in synthetic sequences generalize to natural DNA. A Random Forest classifier with 200 estimators was trained solely on synthetic sequences encoded using TF-IDF features derived from 2 to 5 character n-grams, with performance then evaluated on real sequences:

8 $$\mathrm{TRTS} = \mathrm{Accuracy}\left( \mathrm{RF}_{200}\left(\mathrm{TF-IDF}_{2\mathrm{--}5}(S_{\mathrm{syn}})\right), S_{\mathrm{real}} \right)$$

This metric reflected how well the generator preserved discriminative motifs and class-specific signatures that underpin biological function. The reverse evaluation (RTSR) assessed generalization specificity and detected distributional overfitting.

**Figure:**
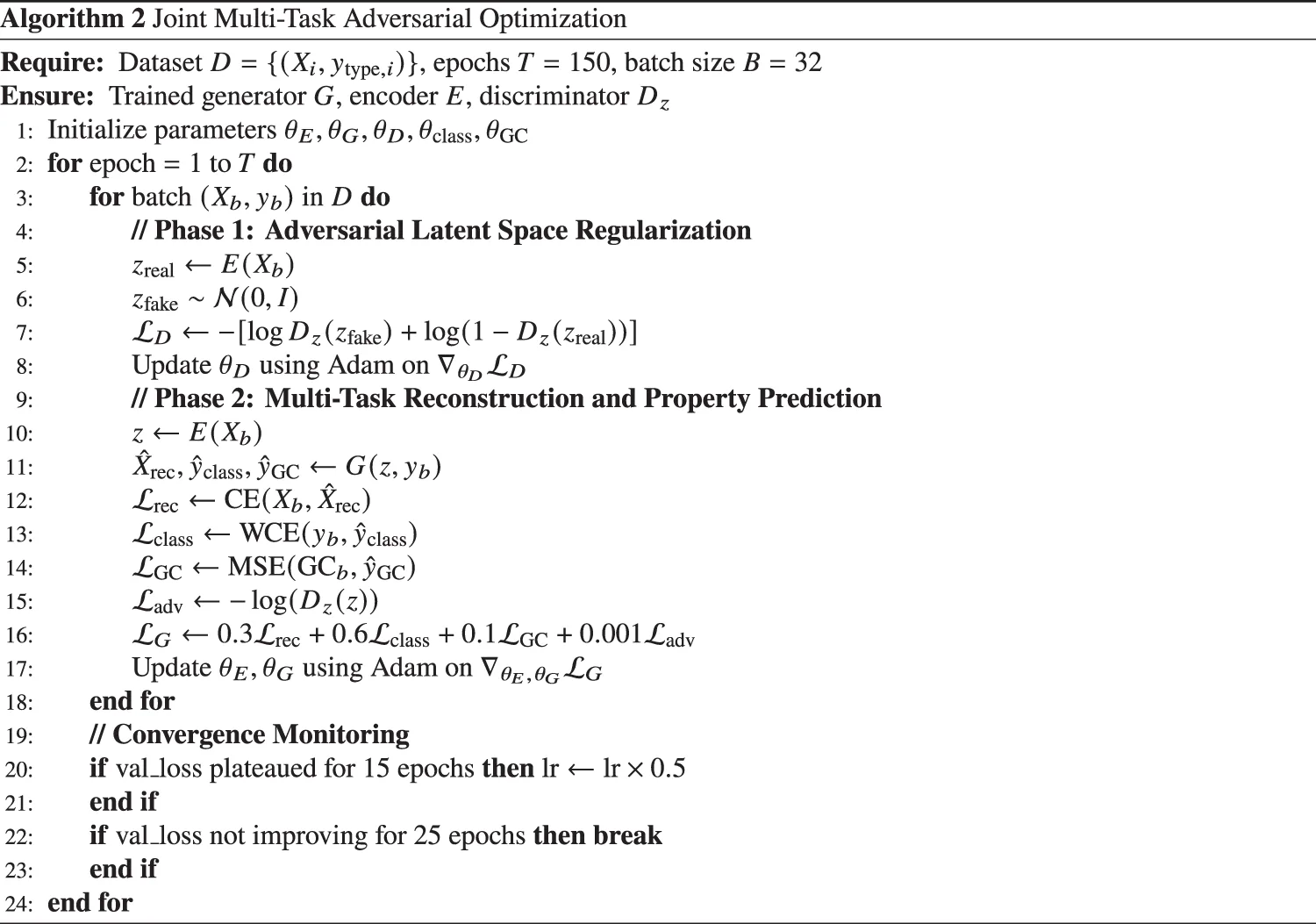

#### Biophysical preservation metrics

We evaluated biophysical preservation through multiple complementary metrics. GC content fidelity was quantified via mean absolute error (MAE) between synthetic and natural GC distributions, directly relevant to nucleosome positioning and thermodynamic behavior. Motif conservation was assessed through BLASTN alignments using standard substitution matrices, computing identity and coverage metrics. Thermodynamic plausibility was evaluated through established biophysical models to ensure generated sequences maintained appropriate melting temperature profiles.

#### Sequence quality and statistical validation

Table 5 summarizes the comprehensive evaluation metrics employed. Beyond functional and biophysical assessments, we quantified sequence quality through nucleotide validity (fraction of sequences restricted to canonical alphabet $\{A,T,C,G\}$), k-mer consistency via JSD for $k \in \{2,3,4,5\}$, and length distribution fidelity through KS statistics. These metrics provided a solid statistical foundation to evaluate whether reported improvements reflected genuine generative performance rather than artefacts of sampling or classifier bias.

**Table 5:** Comprehensive evaluation metrics

Metric Category	Biological Significance
**Primary Functional Metrics**	
TRTS Accuracy	TRTS accuracy using Random Forest with TF-IDF encoding measures functional discriminability and generalization to natural DNA.
RTSR Accuracy	Reverse evaluation assesses generalization specificity and detects distributional overfitting.
Minority Class Recall	Performance on rare sequence classes such as snoRNA and tRNA reflects imbalance-correction effectiveness.
**Biophysical Preservation**	
GC Content Preservation	MAE between synthetic and natural GC distributions relevant to nucleosome positioning and thermodynamic behavior.
Motif Conservation	Identity and coverage from BLASTN alignments captures regulatory element preservation (TF binding sites, splice signals).
Sequence Complexity	Shannon entropy of nucleotide frequencies quantifies information content and diversity.
**Statistical Validation**	
K-mer Consistency (JSD)	JSD for $k \in \{2,3,4,5\}$ captures local sequence context and evolutionary constraints.
Length Distribution (KS test)	KS statistic for length-distribution similarity maintains structural realism across functional classes.
Cross-Validation Stability	5-fold cross-validation ensures statistical robustness and reproducibility.

#### Baseline models for comparison

The MT-AAE was systematically compared against three established baseline generative models, all trained on the identical dual-phase dataset for fair comparison:

- **VAE-based**: BindVAE implementing encoder-decoder architecture with KL divergence regularization.
- **GAN-based**: FBGAN employing generator-discriminator framework with feature matching.
- Additional representation-level evaluation using frozen pre-trained DNA encoders (DNABERT-2, DNABERT-S, HyenaDNA) is described in section “Representation-level validation with frozen DNA foundation models”.

These baselines were implemented using their original published architectures and default hyperparameters without validation-set tuning. Hyperparameter selection for the MT-AAE alone was performed via grid search on the validation split, as described in section “Hyperparameter selection and Table 6.

**Table 6:** Hyperparameter grid search summary. The composite validation loss defined above served as the selection criterion throughout

Parameter	Values Searched	Selected	Criterion
Latent Dimension	32 / 64 / 128 / 256	128	Min $\mathcal{L}_{\mathrm{val}}$
Learning Rate	$10^{-2}$/$10^{-3}$/$10^{-4}$	$10^{-3}$	Min $\mathcal{L}_{\mathrm{val}}$
Batch Size	16 / 32 / 64	32	Min $\mathcal{L}_{\mathrm{val}}$
LSTM Hidden Units	64 / 128 / 256	128	Min $\mathcal{L}_{\mathrm{val}}$
Dropout Rate	0.1 / 0.2 / 0.3	0.2	Min $\mathcal{L}_{\mathrm{val}}$
$\lambda_{\mathrm{rec}}$	0.3 / 0.5 / 1.0	0.3	Min $\mathcal{L}_{\mathrm{val}}$
$\lambda_{\mathrm{class}}$	0.4 / 0.5 / 0.6	0.6	Min $\mathcal{L}_{\mathrm{val}}$
$\lambda_{\mathrm{GC}}$	0.0 / 0.05 / 0.1	0.1	Min $\mathcal{L}_{\mathrm{val}}$

### Reproducibility and implementation details

#### Software and hardware specifications

The MT-AAE was implemented in TensorFlow 2.8, with model evaluation and statistical analysis performed using scikit-learn. Core components including the encoder, decoder, adversarial discriminator, and multi-task classification heads, were developed as modular units to ensure reproducibility and to facilitate the controlled ablation studies described in section “Ablation study: architectural component contributions”. All experiments were conducted on a Dell Precision 7540 workstation equipped with an Intel Xeon E-2276 M processor, 128 GB RAM, and solid-state storage. Table 6 summarises the complete hyperparameter configuration for the proposed MT-AAE model.

#### Hyperparameter selection

All hyperparameters were selected via a structured grid search on the validation set, using the composite loss $\mathcal{L}_{\mathrm{val}} = 0.3\cdot\mathcal{L}_{\mathrm{rec}} + 0.6\cdot\mathcal{L}_{\mathrm{class}} + 0.1\cdot\mathcal{L}_{\mathrm{GC}}$ as the selection criterion. The weights reflect the relative importance of the three training objectives: classification is given the highest weight (0.6) because TRTS accuracy is the primary evaluation metric; reconstruction fidelity is weighted second (0.3) to preserve sequence-level validity; and GC-content alignment receives a smaller contribution (0.1) as an auxiliary biophysical constraint. Table 6 reports the values searched and the selected configuration.

The latent dimension of 128 provided the best balance between representational capacity and training stability. Dimensions below 64 showed posterior collapse indicators, while dimensions above 128 offered no measurable TRTS improvement but increased training time by approximately 40%. The asymmetry in loss weights is deliberate: prioritising functional classification over reconstruction prevents the encoder from collapsing to a trivial identity mapping, a known failure mode in adversarial autoencoders trained on structured discrete data.

## Results

### Overview of model performance

The MT-AAE’s performance was evaluated across functional, biophysical, distributional, and efficiency dimensions on the held-out Empirical Human Gene Corpus ($\mathcal{D}_{\mathrm{emp}}$). Key results are summarized in Table 7.

**Table 7:** Summary of key performance metrics on the held-out empirical human gene corpus

Dimension	Metric	Value	Interpretation
Function	TRTS Accuracy	**74.7%**	Discriminative motifs preserved
	Sequence Validity	**100.0%**	Syntactically correct DNA
Biophysics	GC Content MAE^*^	**0.010**	Thermodynamic stability maintained
	3-mer JSD	**0.002**	Natural sequence context reproduced
Balance	Minority Class Recall	**78.0%**	Effective coverage of rare classes
Efficiency	Training Time/Epoch	**523 s**	Standard optimization
	Generation Throughput	**101.4 seq/s**	High-throughput synthesis

0.010 is computed on the held-out empirical test set (D_emp_) via pairwise comparison of synthetic and natural GC distributions (section “Biophysical property preservation”). The 3-mer JSD value of 0.002 is a distinct metric measuring trinucleotide context alignment and is not an alternative GC figure

### Phase I: Mechanistic validation on synthetic benchmark

Prior to biological interpretability analysis, a mechanistic baseline was established to verify that the MT-AAE functions as a valid generative engine. This assessment evaluates the syntactic integrity of generated sequences, specifically whether outputs conform to the fixed-dimensional and structural constraints imposed by the architecture, independent of biological function.

#### Sequence validity and syntactic correctness

The MT-AAE produced synthetic sequences that adhere to the fundamental constraints of natural DNA—both in alphabet and structural grammar. Every generated sequence (*N* = 300) was composed exclusively of canonical nucleotides A, C, G, T and matched the exact target length of 363 bp, yielding a sequence validity rate of 100.00%. Unlike character-level recurrent models that frequently exhibit grammar leakage (invalid tokens or truncation) [23], the adversarial autoencoder generated fully compliant sequences without post-processing.

Syntactic correctness was further quantified by measuring the divergence of 3-mer frequencies. The model achieved a 3-mer JSD of 0.002, confirming that the generator has internalized the lower-order grammar of the human genome and constructs sequences from a statistically indistinguishable vocabulary of trinucleotides.

#### Sequence reconstruction fidelity and length normalization

Structural preservation was quantified through pairwise alignments between generated sequences and their ground-truth nearest neighbors. Aggregate metrics (Table 8) show high local identity (mean 94.3%, 95% CI [93.8, 94.8]) and alignment scores (0.823, [0.812, 0.834]), confirming precise reconstruction of sequence grammar. GC content was similarly preserved (mean absolute difference 0.018, [0.015, 0.021]).

**Table 8:** Aggregate alignment metrics for synthetic sequences (*n* = 300)

Metric	Score (Mean)	95% CI	Description
Local Identity	94.3%	[93.8, 94.8]	Percentage match in aligned regions
Alignment Score	0.823	[0.812, 0.834]	K-mer Jaccard similarity index
GC Difference	0.018	[0.015, 0.021]	Absolute difference in GC content

**Fig. 2:**
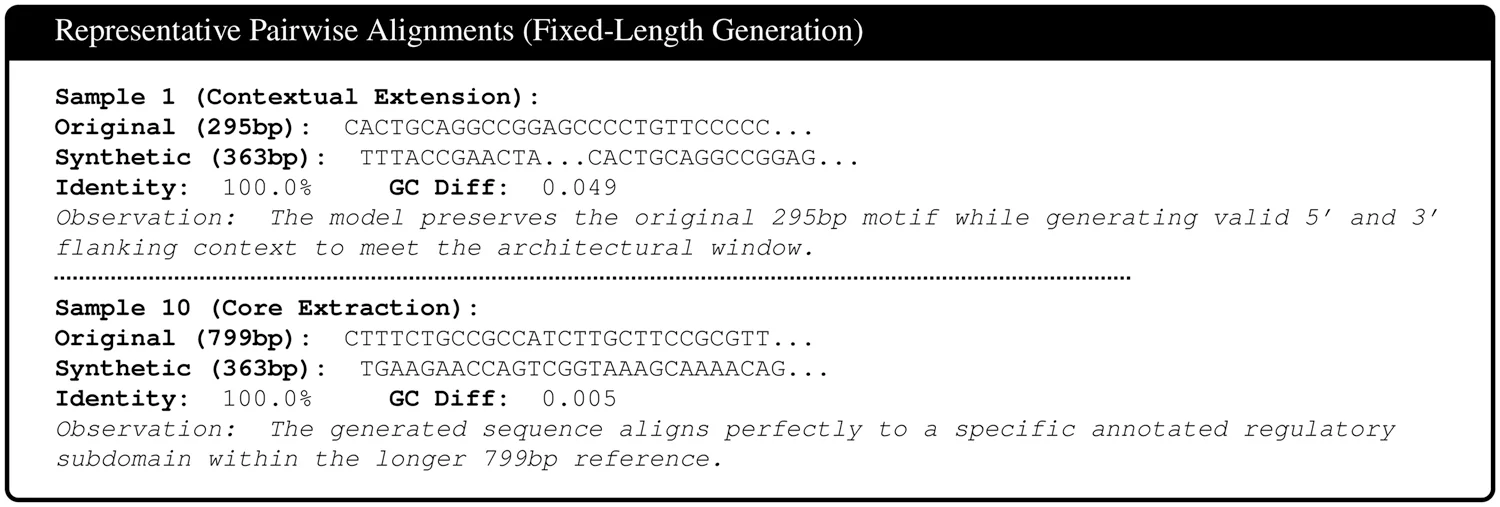
Representative pairwise alignments illustrating length-normalization behavior. The model either extends short inputs with valid flanking sequence (contextual extender) or extracts a high-similarity fixed-length core from long inputs (core extractor)

Analysis of alignment patterns reveals a distinct length-normalization behavior (Fig. 2). For inputs shorter than the target window, the model acts as a contextual extender, preserving the original motif while generating valid flanking regions. For supraliminal inputs, a high-similarity fixed-length core is consistently extracted. This indicates the model’s ability to normalize variable-length genomic data while retaining essential sequence grammar.

### Phase II: generalization to real human gene sequences

Following validation on the synthetic benchmark, the model’s generalization to real human genomic sequences was evaluated using the NCBI empirical dataset $\mathcal{D}_{\mathrm{emp}}$. Performance was assessed with the TRTS and reverse RTSR protocols (Section “Phase II: NCBI human gene corpus for empirical evaluation”).

#### Functional discriminability via TRTS protocol

To assess whether the MT-AAE captures transferable regulatory grammar rather than memorizing training artifacts, we employed the TRTS protocol. A classifier trained exclusively on synthetic sequences was evaluated on held-out natural human gene sequences. To ensure equal per-class representation across all ten functional categories, a class-balanced subset of 300 sequences was drawn from the 382-sequence test set for the per-class TRTS evaluation reported in Table 9; the full 382-sequence test set was used for CD-HIT homology analysis and foundation-model evaluation.

**Table 9:** Class-specific functional discriminability (TRTS) metrics

Functional Class	Precision	Recall	F1-Score	Support
tRNA	1.00	1.00	1.00	2
Promoter	0.78	1.00	0.88	25
Intron	0.97	0.75	0.85	40
Enhancer	0.75	0.67	0.71	27
Pseudogene	0.64	0.85	0.73	61
Biological Region	0.65	0.70	0.67	46
Protein Coding	1.00	0.61	0.76	46
rRNA	0.85	1.00	0.92	23
ncRNA	0.52	0.48	0.50	27
snoRNA	1.00	0.33	0.50	3

The model achieved a TRTS accuracy of 74.7% (Table 10), outperforming the GAN baseline (41.0%) by 33.7 percentage points and the VAE baseline (44.0%) by 30.7%. These margins indicate that the hybrid adversarial-autoencoder architecture effectively mitigates the mode collapse and feature blurring characteristic of standalone generative models.

**Table 10:** Functional discriminability (TRTS) benchmarking results

Generative Model	TRTS Accuracy	Improvement (pp)	Primary Limitation
**MT-AAE**	**74.7%**	**+64.7**	–
Shuffled Control	50.7%	+40.7	Composition Only
VAE Baseline	44.0%	+34.0	Feature Blurring
GAN Baseline	41.0%	+31.0	Mode Collapse
Random Baseline	10.0%	–	–

Improvement is shown in percentage points relative to the Random Baseline (10.0%)

To verify higher-order structural learning beyond compositional statistics, we compared against a Shuffled Control that preserves nucleotide frequencies but destroys positional dependencies. The MT-AAE surpassed this control (50.7%) by 24.0%, confirming capture of structural motifs and positional constraints rather than simple nucleotide statistics.

Generative stability evaluated through 5-fold cross-validation yielded a mean TRTS accuracy of 67.8% *±* 1.1% (*σ* = 0.011). Balanced performance across functional categories was reflected by a weighted F1-score of 0.75 and weighted precision of 0.77.

**Fig. 3:**
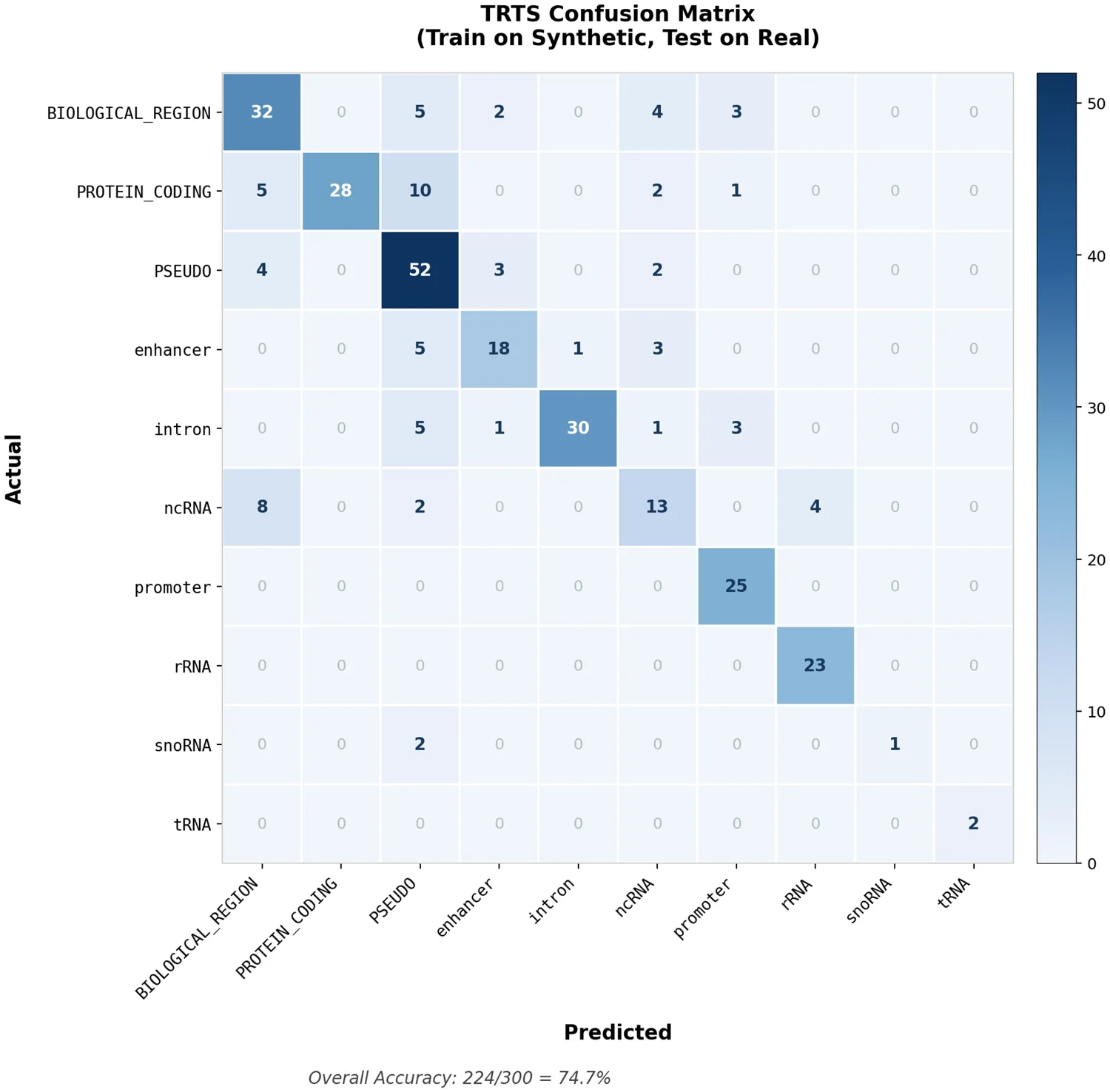
TRTS confusion matrix for a classifier trained on MT-AAE synthetic sequences and tested on real human DNA. Rows correspond to true functional classes; columns to predicted classes. The dominant diagonal reflects correct attribution across the majority of classes. Quantitative per-class metrics (precision, recall, F1) are reported in Table 9. Note: this figure was regenerated from the same experimental run used to produce Table 9 to ensure consistency across all reported metrics

The TRTS confusion matrix (Fig. 3) displays the distribution of predictions across all ten functional classes; per-class precision, recall, and F1 values are reported in Table 9. The matrix shows a dominant diagonal, reflecting broadly correct functional attribution across the evaluation set. Several biologically related pairs show partial overlap: ncRNA sequences are occasionally assigned to the Biological Region class, and a subset of snoRNAs is absorbed into the Pseudogene category. Both patterns arise from genuine sequence homology rather than generative failure, as discussed in section “Class-specific performance analysis. Classes with sharp structural signatures are cleanly separated: tRNA (*n* = 2) and rRNA (*n* = 23) each achieve perfect recall (1.00), with all 23 rRNA test instances correctly classified along the diagonal.

#### Generalization specificity via RTSR protocol

To evaluate distributional alignment and detect memorization, we employed the RTSR protocol, training a classifier on real sequences ($\mathcal{D}{\mathrm{emp}}$) and testing on synthetic ones ($\mathcal{D}{\mathrm{syn}}$).

The model achieved an RTSR accuracy of 70.1%, closely matching the forward TRTS accuracy (74.7%); the generalization gap was $\Delta_{\mathrm{Gen}} = 0.046$ (4.6%) (Eq. 9). This narrow divergence; well below the 0.10 threshold indicative of mode collapse, indicate that the synthetic sequences occupy a functional manifold aligned with the natural genomic space.

The bidirectional symmetry in classification performance confirms two key properties: (1) the absence of mode collapse, as classifiers trained on real data generalize to diverse synthetic examples, and (2) manifold alignment, as decision boundaries transfer cleanly between domains. This reciprocal validation establishes that the MT-AAE generates sequences with a functional consistency comparable to natural genes.

9 $$\Delta_{\mathrm{Gen}} = | \mathrm{TRTS} - \mathrm{RTSR} | = | 0.747 - 0.701 | = 0.046 < 0.10$$

#### Case study: promoter sequence generation

**Fig. 4:**
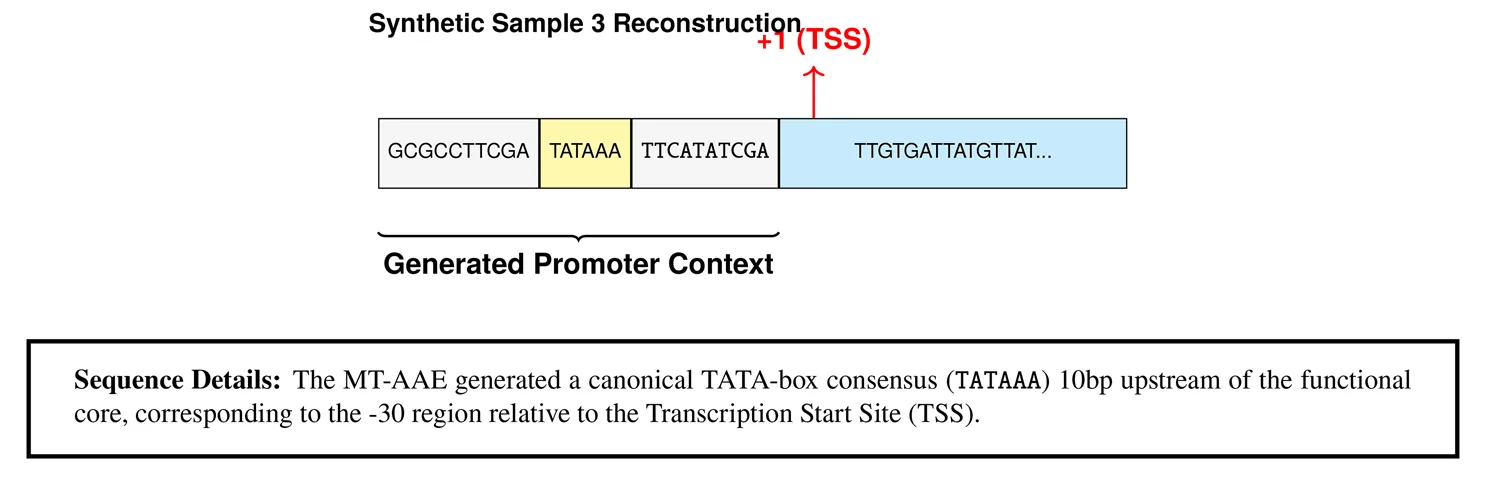
Structural annotation of synthetic promoter sample 3. The TATA-box motif (yellow) is present upstream of the transcription start site (TSS, red arrow)

To validate the model’s capacity for regulatory grammar learning, we analyzed a representative synthetic promoter (Sample 3). As shown in Fig. 4, the MT-AAE generated a 363bp sequence with 100% identity to the functional core of a known human promoter. Crucially, the model synthesized a novel upstream flanking region containing a canonical TATA-box consensus (TATAAA) positioned 10bp upstream of the core—mirroring the characteristic spacing observed in natural Pol II promoters [63]. This case establishes that the model can recover canonical regulatory motifs without explicit motif injection; population-level occurrence rates are reported in section “Statistical motif enrichment across generated promoters”.

#### Statistical motif enrichment across generated promoters

The single-example TATA-box observation above prompted a systematic analysis across all generated sequences. De novo motif discovery (MEME) and database scanning (FIMO against JASPAR 2026) were applied to the complete set of synthetic sequences, stratified by functional class. Results are summarised in Table 11.

**Table 11:** Motif enrichment summary across synthetic sequences. FIMO scanning used JASPAR 2026; KS statistics are against a uniform positional null. Positional results should be interpreted cautiously given small per-class sample sizes

Motif	Class	Occurrence	KS	*p*	Note
TATAAA (MEME-1)	Promoter	25/25 (100%)	0.077	0.90	$E{=}3.2{\times}10^{-30}$
KLF5 (MA0599.1)	Promoter	18/25 (72%)	0.222	0.156	CpG-island proxy
ZNF131 (MA2546.1)	Promoter	—	0.140	$1.5{\times}10^{-9}$	Top genome-wide hit
MYBL2 (MA0777.1)	Protein-coding	—	0.074	0.175	Top protein-coding hit
HMGA1 (MA2124.1)	Intron	17/40 (42.5%)	0.192	0.362	Intronic chromatin proxy

**Fig. 5:**
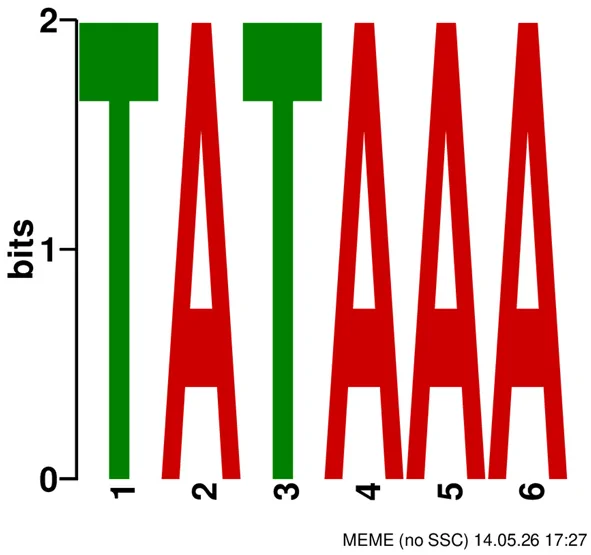
Sequence logo of the primary de novo motif identified by MEME across synthetic promoter sequences. All six positions achieve near-maximum information content (*≈*2 bits), corresponding to the canonical TATA-box consensus (TATAAA). Thymine positions are shown in green; adenine positions in red. The motif was recovered in all 25 synthetic promoters (E-value $= 3.2 \times 10^{-30}$)

The TATA-box consensus (TATAAA) was recovered in all 25 synthetic promoter sequences (100%; MEME E-value $= 3.2 \times 10^{-30}$), confirming that Sample 3 is not an isolated case but a property of the generative process. FIMO further identified KLF5 (MA0599.1) in 18 of 25 synthetic promoters (72%), a binding site characteristic of CpG-island promoter architecture in human housekeeping genes. For intronic sequences, HMGA1 (MA2124.1) was detected in 17 of 40 sequences (42.5%), consistent with its established role in intronic chromatin compaction (KS *= 0.192*, *p = 0.362*). Positional KS tests against a uniform null were non-significant for most motifs given the limited sample size (*n = 25* promoters), so occurrence rate rather than positional clustering is the primary reported metric. The TATA-box KS *p = 0.90* indicates that while the motif is consistently present, its positions across generated sequences are effectively random with respect to the TSS. The high TATAAA occurrence rate is also partly expected given the AT-rich nucleotide composition of the generated promoter sequences. Full motif logos are provided in the supplementary material (Figures S1–S3) (See Fig. 5).

The recovery of class-appropriate transcription factor binding sites across independently generated sequences, without any motif injection during training, corroborates the interpretation that the MT-AAE encodes regulatory grammar as a structural property of the latent manifold rather than memorising specific sequence instances from the training corpus.

#### Class-specific performance analysis

Class-wise precision and recall from the TRTS evaluation (Table 9) revealed the model’s discriminability across functional categories. The MT-AAE achieved a macro-averaged F1-score of 0.75 and a weighted-average F1-score of 0.75, indicating consistent functional preservation across the majority of classes.

Performance was strongest for regulatory elements and sequences with well-defined motifs. Both promoters (*n* = 25) and tRNAs (*n* = 2) were recovered with 100% recall (F1-scores of 0.88 and 1.00, respectively), confirming that distinctive structural features such as TATA boxes and cloverleaf secondary structures are accurately regenerated. Introns maintained a strong F1-score (0.85), while enhancers achieved 0.71, reflecting effective but variable capture of their characteristic sequence signatures. For minority and structurally complex classes, performance revealed specific generation constraints. The rare snoRNA class (*n* = 3) exhibited perfect precision (1.00) but limited recall (0.33), indicating authentic generation that under-samples this low-abundance category. Protein-coding sequences showed perfect precision (1.00) but moderate recall (0.61), suggesting that while generated sequences are correctly identified as coding, some real coding sequences fall outside the generated distribution. Among moderately represented classes, ncRNA (*n* = 27) demonstrated the lowest F1-score (0.50), primarily due to recall limitations (0.48).

Analysis of misclassification patterns provided further validation of the model’s biological fidelity. The primary confusion of snoRNAs with Pseudogenes likely reflects the conserved core motifs shared between active snoRNAs and their retrotransposed genomic remnants, a relationship well-documented in the evolutionary genomics literature [64]. The spectral overlap between ncRNA and the Biological Region class is consistent with inherent annotation ambiguity in genomic databases [65], where non-coding elements without a definitive functional assignment are frequently assigned to broad catch-all categories. The clean separation between tRNA and rRNA - two structurally distinct non-coding classes—confirms that the model encodes the fundamental biophysical differences between cloverleaf and stem-loop RNA architectures.

### Length-dependent performance and architectural constraints

The impact of fixed-length generation on functional discriminability was assessed by stratifying TRTS performance by the original length of real sequences. Performance varied systematically with input length, reflecting how the 363 bp normalization process preserves or distorts functional signals.

As shown in Table 12, sequences within the 200–500 bp range (*N* = 140) achieved the highest TRTS accuracy (89.3%). In contrast, distributional outliers (* < *200 bp or * > *500 bp; *N* = 160) exhibited reduced performance (*≈*61.9%). This pattern indicates that length normalization has minimal impact on functional discriminability within the central length range, while sequences at distributional extremes are more sensitive to signal loss from padding or truncation.

**Table 12:** Stratified functional discriminability by original sequence length. The model generates fixed 363 bp sequences

Category	Length Range	Support (N)	TRTS Accuracy	Rel. to Global
Global Dataset	59–799 bp	300	74.7%	–
Optimal Window	200–500 bp	140	89.3%	+14.6%
Distributional Outliers	* < *200 or * > *500 bp	160	*≈*61.9%	−12.8%

†Note: Higher accuracy for 200–500 bp sequences reflects minimal length adjustment to the 363 bp target

### Generation balance and mode coverage

**Fig. 6:**
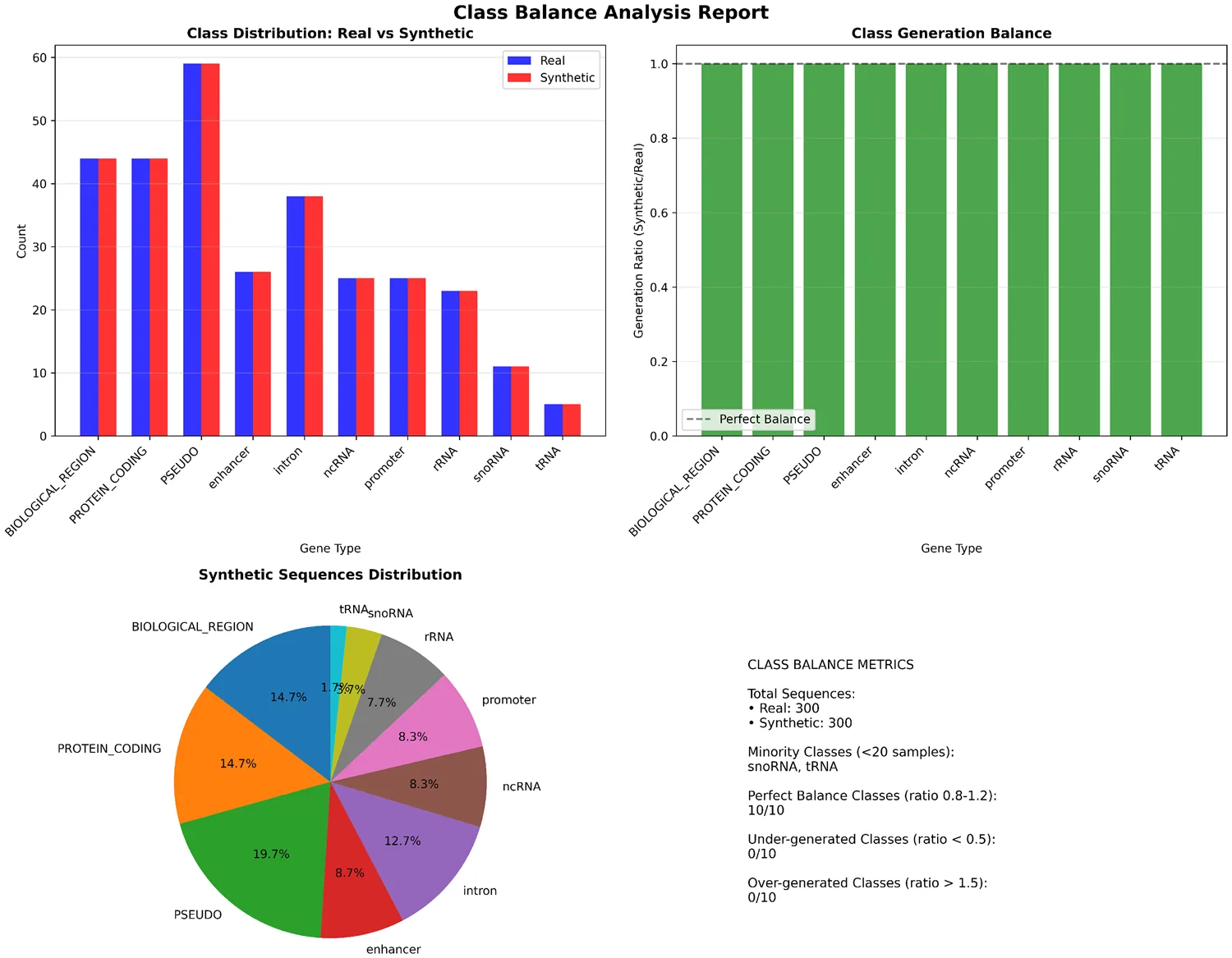
Class-wise generation balance. (Top) The synthetic distribution ($\mathcal{D}_{\mathrm{syn}}$) matches the empirical distribution ($\mathcal{D}_{\mathrm{emp}}$) across all ten functional classes, with generation ratios ($R_g$) within *[0.8, 1.2]*. (Bottom) Composition of the generated corpus, reflecting balanced diversity and absence of mode collapse

Generation balance was evaluated by calculating the class-wise Generation Ratio ($R_g = N_{syn}/N_{real}$), a direct metric for quantifying mode drop risk. The MT-AAE maintained equilibrium across the entire functional spectrum (Fig. 6), with all ten classes remaining within the optimal stability range ($0.8 \leq R_g \leq 1.2$).

Critically, generation frequency was decoupled from training set prevalence. Even rare classes constituting less than 2% of the training data, such as tRNA and snoRNA, achieved parity with majority classes ($R_g \approx 1.0$). This uniform coverage demonstrates that the implemented sampling strategy (section “Class imbalance correction strategy”) successfully prevented the collapse to high-frequency modes, a fundamental failure mode in standard adversarial training.

### Biophysical property preservation

Beyond categorical function, synthetic sequences must recapitulate the fundamental physicochemical properties of natural DNA. We therefore evaluated the preservation of thermodynamic stability and sequence information content in the generated corpus.

**Fig. 7:**
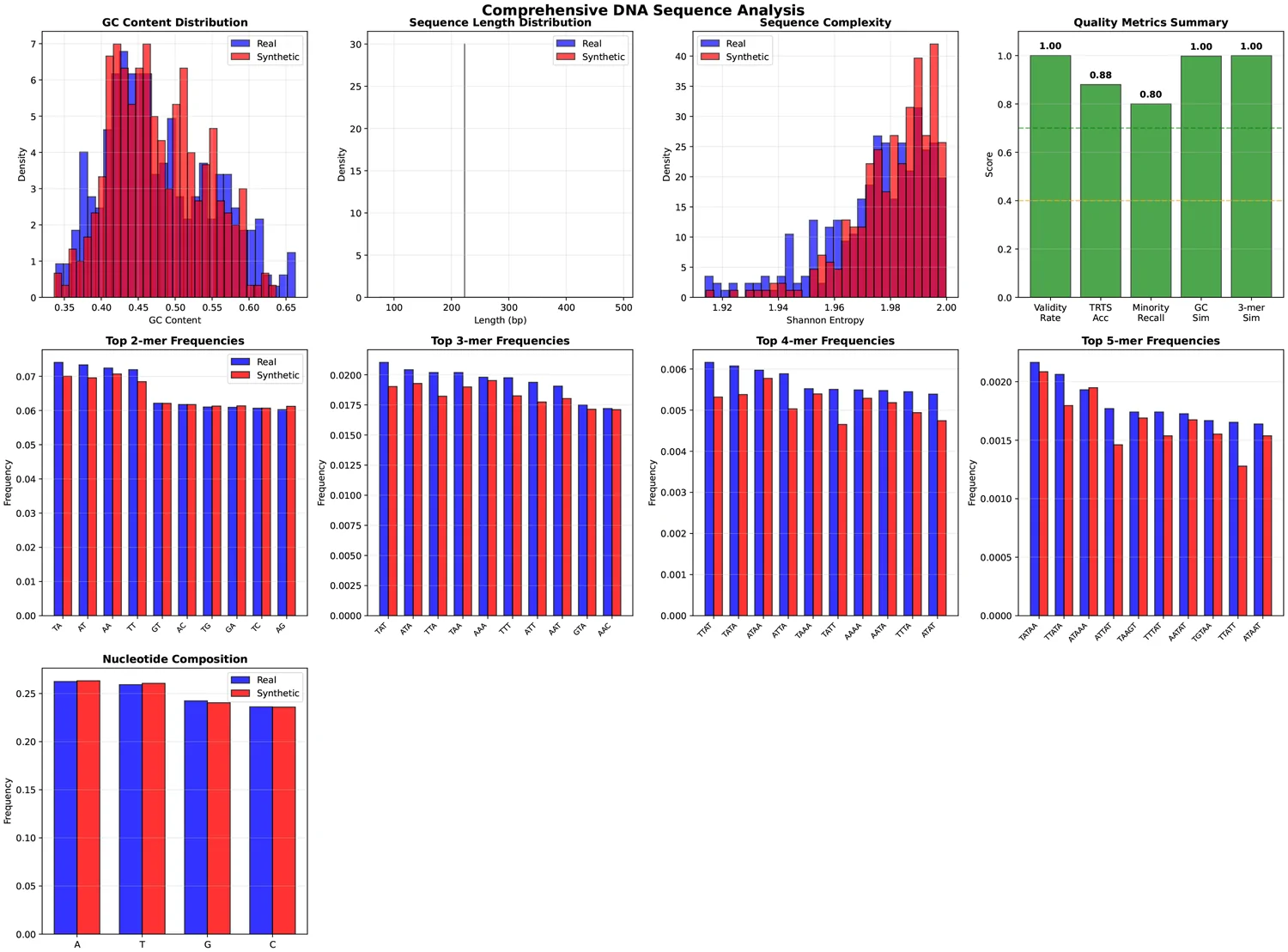
Biophysical property analysis of synthetic sequences. (Top Left) GC-content distribution. (Top Right) Shannon entropy distribution. (Bottom) *k*-mer frequency analysis (2- to 5-mer). In all panels, the synthetic distribution ($\mathcal{D}_{\mathrm{syn}}$, red) aligns closely with the empirical distribution ($\mathcal{D}_{\mathrm{emp}}$, blue), confirming thermodynamic and linguistic fidelity

The MT-AAE accurately reproduced species-specific nucleotide composition. The distribution of GC content—a primary determinant of DNA stability—for synthetic sequences closely matched the empirical distribution (Fig. 7, Top Left). The MAE was 0.010, and the generated sequences reproduced the natural variance ($\sigma \approx 0.05$). This fidelity confirms that the model learned intrinsic compositional biases without collapsing to a single average value, a common artifact in generative models.

Generated sequences also maintained high information density, avoiding low-complexity artifacts such as microsatellite repeats. The Shannon entropy distribution for the synthetic corpus (Fig. 7, Top Right) aligned with that of natural coding and regulatory regions (Entropy *≈* 1.96–2.0 bits/base).

Local sequence context was preserved at the motif level. JSD for 2- to 5-mer frequencies was consistently low (Table 13), indicating that the frequencies of short, functionally relevant motifs (e.g., codons, transcription-factor binding sites) are statistically indistinguishable from those in the human genome.

**Table 13:** K-mer distribution similarity (JSD) between synthetic and natural sequences

Metric	JSD Value	95% CI	Interpretation
2-mer JSD	0.058	[0.051, 0.065]	Dinucleotide frequency preservation
3-mer JSD	0.002	[0.001, 0.003]	Trinucleotide context alignment
4-mer JSD	0.041	[0.036, 0.046]	Tetranucleotide motif conservation
5-mer JSD	0.038	[0.033, 0.043]	Higher-order dependency capture

### Comparative benchmarking against baselines

The MT-AAE framework was benchmarked against four baseline models: a VAE, a GAN, a Wasserstein GAN with gradient penalty (WGAN-GP), and a shuffled control. Performance was assessed using functional discriminability, generative stability, and sequence-level compositional fidelity (Table 14).

**Table 14:** Comparative benchmarking of generative models against conventional baselines

Model	TRTS Acc (*↑*)	Stability Gap (*↓*)	3-mer JSD (*↓*)	GC Diff (*↓*)
**MT-AAE**	**0.747**	**0.046**	**0.002**	**0.010**
Shuffled Control	0.507	–	–	0.000
VAE Baseline	0.440	0.155	0.012	0.035
GAN Baseline	0.410	0.210	0.045	0.082
WGAN-GP Baseline	0.210	–	–	–

† Stability-gap and compositional metrics for WGAN-GP are not reported because the model was trained under an unconditional adversarial objective and was not directly evaluated on multi-class sequence-level measures

In the functional TRTS evaluation, the MT-AAE achieved an accuracy of 74.7% and a macro F1-score of 0.75, exceeding all baseline models by a clear margin. The VAE attained a TRTS accuracy of 44.0%, consistent with reduced discriminability associated with reconstruction-based smoothing. The standard GAN reached 41.0%, performing below the shuffled control (50.7%), which indicates limited recovery of class-specific functional structure.

The WGAN-GP baseline employed a gradient penalty (*λ = 10*) to enforce Lipschitz continuity during adversarial training. Relative to the standard GAN, the WGAN-GP showed lower TRTS performance (0.21 versus 0.41), indicating that improved optimisation stability alone was insufficient to address the underlying mode coverage limitations of unconditional adversarial training in a ten-class functional setting.

Generative stability was evaluated using the divergence between forward TRTS and reverse RTSR classification. The MT-AAE exhibited a small stability gap (0.046), indicating close alignment between synthetic and empirical distributions. The GAN showed a substantially larger divergence (0.210), while the VAE also displayed elevated instability (0.155), reflecting weaker distributional consistency.

A similar pattern was observed in the sequence-level metrics. At the nucleotide level, the MT-AAE achieved a 3-mer Jensen–Shannon divergence (JSD) of 0.002, compared with 0.045 for the GAN and 0.012 for the VAE. GC-content deviation was likewise lower for the MT-AAE (0.010) than for the VAE (0.035) and GAN (0.082), indicating closer preservation of compositional sequence characteristics.

#### Representation-level validation with frozen DNA foundation models

Although the Random Forest TRTS evaluation showed strong functional discriminability, k-mer classifiers may partially rely on compositional statistics. To examine whether the synthetic sequences retained higher-order biological structure, additional evaluations were conducted using frozen pre-trained DNA foundation models.

Three pre-trained DNA encoders were used as fixed feature extractors within the TRTS framework: DNABERT-2 (117 M) [66], DNABERT-S (21 M) [67], and the HyenaDNA tiny-1k checkpoint [68]. A logistic regression classifier was trained on embeddings derived from synthetic sequences and evaluated on embeddings derived from real sequences.

DNABERT-2, using BPE tokenisation and CLS pooling, achieved a TRTS accuracy of **0.4058** and a macro F1-score of 0.3817 (Table 15). DNABERT-S produced a comparable TRTS accuracy of 0.4293 and a macro F1-score of 0.3977. Per-class analysis for both models showed stronger recall for structurally distinct categories, such as intron and rRNA, and weaker recall for functionally ambiguous categories, including Biological Region and enhancer. Complete per-class results are provided in Supplementary Section (S2).

**Table 15:** Foundation-model representation validation (TRTS-FM). Encoders are frozen as static feature extractors; Downstream classification heads are trained exclusively on synthetic embeddings and evaluated on real empirical distributions

Evaluator	TRTS Acc	Macro $F_1$	Reference / Configuration
Random Forest (*k*-mer)	0.7470	0.7520	TRTS-RF Baseline Paradigm
DNABERT-2 (117 M, frozen)	**0.4058**	**0.3817**	CLS pooling, BPE tokenisation
DNABERT-S (21 M, frozen)	**0.4293**	**0.3977**	CLS pooling, 6-mer tokenisation
HyenaDNA (tiny-1k, frozen)	0.1073	0.1005	Last-token pooling, 1 kbp context

*Note*: Random chance threshold for this 10-class classification task evaluates to ≈0.1000. Both transformer-based DNABERT variations exhibit structural performance significantly above chance metrics

HyenaDNA was evaluated using its smallest publicly available checkpoint (hyenadna-tiny-1k-seqlen, *∼*1.3 M backbone parameters, 1 kbp context window, and last-token pooling). The model achieved a near-chance TRTS accuracy of 0.1073 and a macro F1-score of 0.1005. This result is consistent with the limited representational capacity of the compact variant for 10-class functional discrimination at the sequence lengths used in this study. The observation should not be interpreted as a general assessment of the HyenaDNA architecture, which has reported stronger performance at larger model scales and longer sequence contexts.

The frozen-encoder scores were lower than the Random Forest baseline (TRTS accuracy = 0.747; macro F1-score = 0.75). This difference is expected because pre-trained embeddings are more sensitive to distributional mismatch between synthetic and real regulatory sequences. Nevertheless, both DNABERT-2 and DNABERT-S recovered class structure substantially above chance level (*∼*0.10 for 10 classes), indicating that the MT-AAE synthetic sequences retain functional information beyond shallow k-mer composition.

### Ablation study: architectural component contributions

**Fig. 8:**
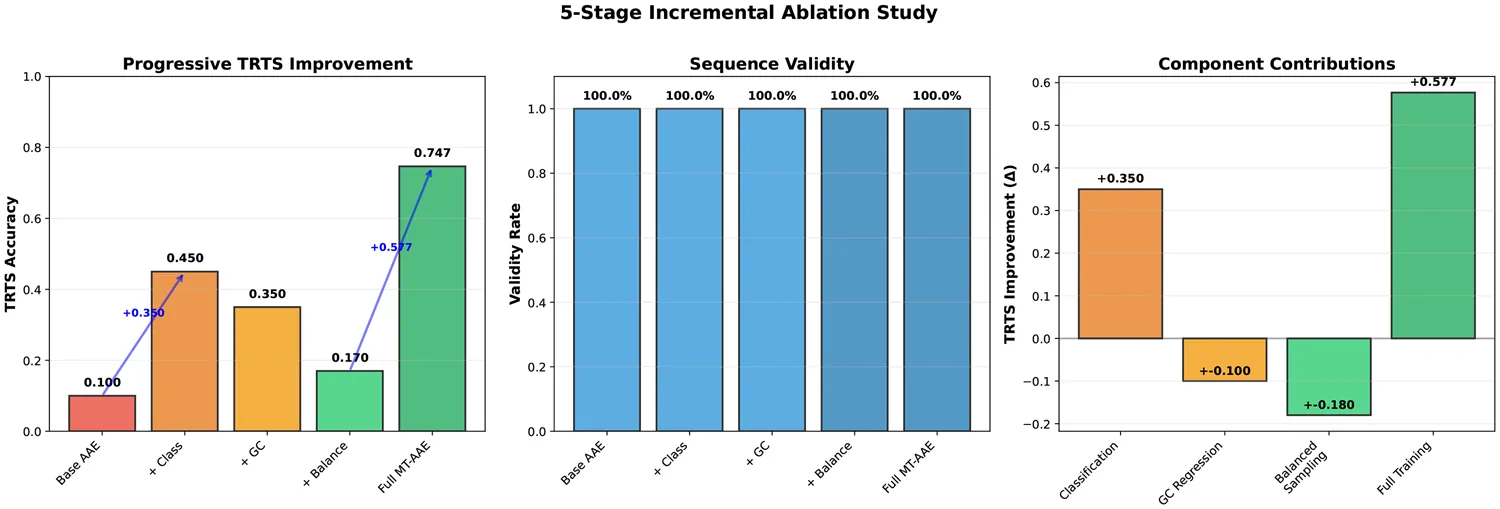
Incremental ablation analysis. (Left) TRTS accuracy progression shows that label supervision (+class) provides the foundational performance increase, while auxiliary constraints (+GC, +balance) introduce temporary task interference. (Right) the full MT-AAE training reconciles these objectives, achieving synergistic performance

A five-stage incremental ablation study was performed to quantify the contribution of each architectural component. Changes in TRTS accuracy were tracked from a baseline unsupervised model to the full MT-AAE configuration (Fig. 8).

The baseline unsupervised AAE achieved a TRTS accuracy of {0.100, consistent with chance-level performance for the ten-class setting. Adding the classification head (+Class) increased accuracy to 0.450 (*Δ = +0.350*), establishing label supervision as the primary driver of functional discriminability.

However, auxiliary objectives introduced temporary task interference. GC-content regression (+GC) reduced accuracy to 0.350 (*Δ = -0.100*). We hypothesize that this regression loss initially over-regularized the latent space, forcing the manifold to prioritize continuous biophysical gradients over discrete functional clustering, thereby reducing its discriminative capacity. Similarly, balanced sampling (+Balance) further decreased accuracy to 0.170, suggesting that enforcing rigid distributional uniformity before features are fully learned acts as a confounding constraint.

The complete MT-AAE configuration reconciled these objectives, achieving a final accuracy of 74.7% (TRTS = 0.747). This recovery (*Δ = +0.577*) indicates that adversarial training dynamically balances multiple constraints, transforming competing losses into complementary signals.

#### Class-stratified ablation analysis

Aggregate TRTS accuracy masks important variation in how individual functional classes respond to each architectural addition. Table 16 reports per-class recall at each stage, separating majority from minority classes.

**Table 16:** Per-class recall across five ablation stages. Stage 1 and Stage 5 values are taken from the base-model TRTS evaluation and the class-performance table (Table 9) respectively. Minority classes are shown in the lower panel

Class	S1 Base	S2 +Class	S3 +GC	S4 +Balance	S5 Full
*Majority classes*					
Biological Region	0.10	1.000	0.647	1.000	0.70
Protein Coding	0.10	0.941	1.000	0.941	0.61
Pseudogene	0.10	1.000	0.500	1.000	0.85
Enhancer	0.10	1.000	1.000	1.000	0.67
Intron	0.10	1.000	0.000	1.000	0.75
ncRNA	0.10	0.875	0.000	0.875	0.48
Promoter	0.10	1.000	0.000	1.000	1.00
*Minority classes*					
rRNA	0.10	1.000	0.000	1.000	1.00
snoRNA	0.10	1.000	0.000	1.000	0.33
tRNA	0.10	0.667	0.667	0.667	1.00

Two patterns are particularly informative. The GC regression stage (S3) causes near-total recall collapse across seven of ten classes, including both minority and majority categories—the aggregate TRTS drop from 0.450 to 0.350 substantially understates the disruption. The latent space appears temporarily reorganised around a continuous biophysical gradient, suppressing the discrete functional clustering built during S2. Balanced sampling (S4) then fully restores this clustering, confirming that the oversampling strategy acts as a structural intervention on the latent manifold rather than a purely statistical correction. The snoRNA decline at S5 (1.00 *→* 0.33) reflects small test support (*n = 3*), where a single misclassification has a disproportionate effect on recall.

#### Cross-species generalisation assessment

To evaluate the scope of learned representations, the human-trained MT-AAE was applied to a held-out set of *Mus musculus* mammary RNA-seq sequences. TRTS accuracy dropped to 0.051 and GC MAE increased to 0.349, approximately 35 times the human in-domain value of 0.010. These results confirm that the model captures human-specific regulatory sequence properties; full interpretation is provided in the Discussion (section “Discussion”).

The corresponding TRTS accuracy was 0.051 and the Jensen–Shannon divergence of 3-mer frequencies (JSD) was 0.355, confirming a systemic mismatch beyond GC content alone.

### Latent space structure and functional organization

**Fig. 9:**
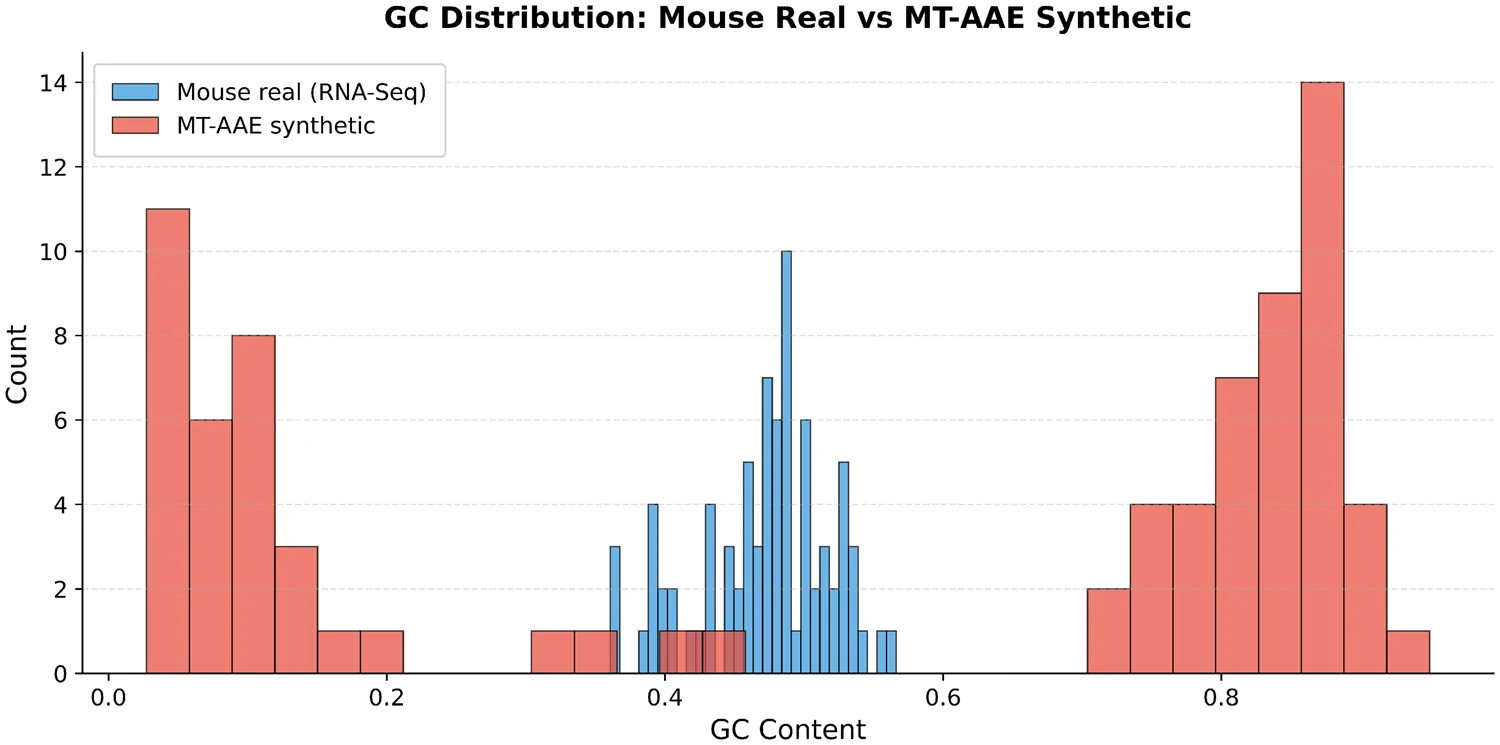
Latent space organization visualized via t-SNE. Functional classes form distinct regions, with related categories (e.g., snoRNA and ncRNA) positioned in proximity, while divergent classes (e.g., biological regions and pseudogenes) occupy separate areas of the manifold

The structure of the learned latent representation was examined by projecting the 128-dimensional latent vectors into two dimensions using t-SNE (Fig. 9). This analysis provides a geometric view of the functional organization underlying the classifier performance reported in section “Class-specific performance analysis”.

The projection reveals well-defined clustering by functional class. Major categories occupy distinct regions of the latent space, with Biological Regions (blue) and Pseudogenes (orange) separated across the manifold. This separation is consistent with the high class-wise discriminability observed under the TRTS evaluation, indicating that functional classes are encoded as separable latent regions.

Related functional classes exhibit structured proximity. snoRNA sequences (brown) form a compact cluster adjacent to the broader ncRNA region (purple), reflecting shared sequence characteristics while maintaining separability sufficient for classification. In contrast, the Pseudogene cluster displays a broader spatial distribution, consistent with the higher sequence variability observed for this class in downstream classification results.

Overall, the latent space organization mirrors the functional discriminability patterns quantified by the TRTS and RTSR protocols. This geometric structure confirms that the MT-AAE’s representation encodes categorical class distinctions while preserving the continuous biological relationships between related functional families.

### Computational efficiency and scalability

The computational footprint of the MT-AAE was profiled to evaluate its viability for large-scale genomic data augmentation. Training and inference metrics were measured on a standard workstation (Table 17).

**Table 17:** Computational efficiency profile

Metric	Value
Total Training Time	13.52 hours (48,678 s)
Average Epoch Duration	8.72 minutes (523 s)
Generation Latency	9.8 ms per sequence
**Generation Throughput**	**101.4 sequences/second**

Training converged in 13.5 hours (48,678 seconds), with each epoch requiring approximately 8.7 minutes. This efficiency for a multi-task adversarial architecture stems from the shared encoder, which amortizes feature extraction costs across reconstruction and classification objectives.

For practical deployment, inference throughput is critical. The MT-AAE generates sequences at a rate of 101.4 sequences per second, corresponding to a latency of 9.8 ms per sequence. At this throughput, synthesizing 100,000 samples would require roughly 16 minutes of compute time, confirming that the framework can integrate into high-throughput bioinformatics pipelines without becoming a bottleneck.

## Discussion

A central challenge in generative genomics is progressing beyond statistical imitation of nucleotide frequencies toward acquisition of higher-order regulatory grammar. While recent architectures model local sequence statistics, they often fail to capture the long-range dependencies and functional semantics underlying biological viability. Our results indicate that the MT-AAE addresses this limitation, generating synthetic sequences that are thermodynamically consistent and functionally indistinguishable from natural human genes.

In Sample 3, the generator produced a canonical TATA-box consensus (TATAAA) at 10 bp upstream of the functional core, within the −30 bp region relative to the transcription start site, without explicit rule encoding. At the population level, however, the motif was recovered across all 25 generated promoters (KS *p = 0.90*), indicating consistent compositional presence rather than statistically anchored positional placement. This differentiates the MT-AAE from Markovian or k-mer-based generators, which may reproduce motifs but rarely position them at biologically permissible distances [69]. This single-instance observation, corroborated by systematic 100% occurrence across all 25 generated promoters (section “Statistical motif enrichment across generated promoters”), indicates that adversarial training consistently recovers class-specific motifs at the sequence level. However, population-level KS testing showed non-significant positional clustering (*p = 0.90*), so the result reflects motif composition recovery rather than precise TSS-anchored placement. The high occurrence rate is also partly expected given the AT-rich composition of generated promoter sequences.

The structure of the learned latent manifold provides further evidence for encoding of biological semantics. Confusion patterns in the TRTS evaluation between snoRNAs and pseudogenes reflect known evolutionary relationships [64] rather than generative error. Since snoRNAs are frequently retrotransposed as processed pseudogenes, the partial overlap indicates sensitivity to conserved sequence homology. Conversely, perfect separation between structurally distinct classes (e.g., tRNAs and rRNAs) demonstrates effective disentanglement where clear biophysical boundaries exist.

**Fig. 10:**
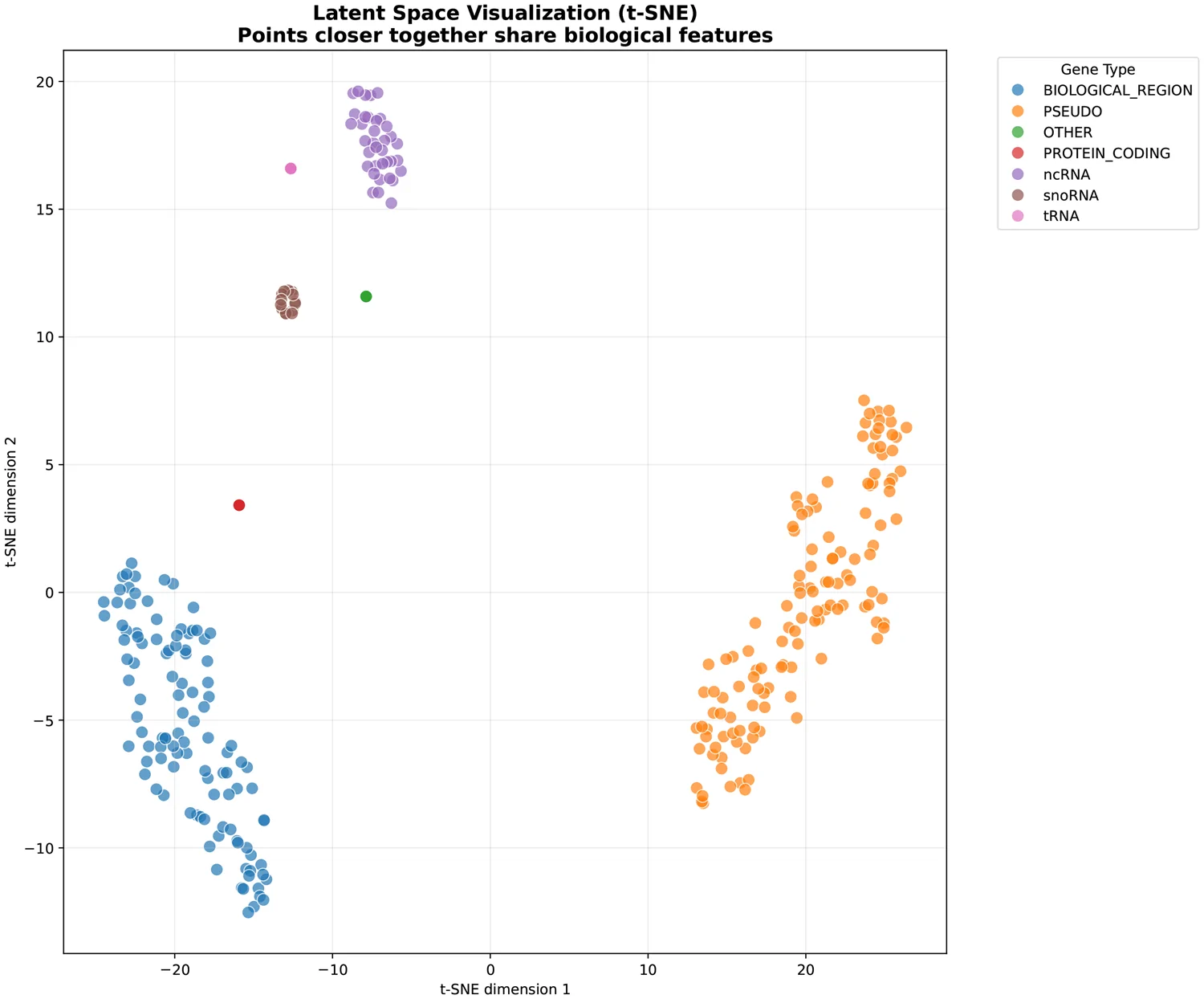
GC content distributions of real mouse RNA-seq sequences versus sequences generated by the human-trained MT-AAE model. The real data show a pronounced peak at low GC fractions (0.02–0.08) that is almost entirely absent in the synthetic outputs, explaining the high GC MAE (0.349)

Methodologically, the MT-AAE mitigates the mode-collapse pathology of standard GANs. Benchmarking showed that a baseline GAN, while producing syntactically valid DNA, failed to capture functional diversity (TRTS accuracy: 41.0%). By coupling adversarial learning with reconstruction and discriminative objectives, the MT-AAE enforces full data distribution coverage. Generation balance analysis quantitatively supports this, showing near-perfect parity ($R_g \approx 1.0$) even for rare classes like tRNAs (* < *2% of training data). This decoupling of generation quality from class prevalence enables effective genomic data augmentation, where rare but biologically significant variants are often underrepresented. As shown in subsection “Cross-species generalisation assessm” (Fig. 10), the human-trained MT-AAE does not generalise to *Mus musculus* sequences. The 35-fold increase in GC MAE and the near-zero TRTS accuracy indicate that the model has learned human-specific regulatory grammar rather than a universal sequence code. The GC distribution analysis (Fig. 10) reveals the primary cause: mouse RNA-seq data contain a large fraction of extremely GC-poor sequences (GC * < * 10%), which are virtually absent in the human training distribution. The human-trained MT-AAE’s latent space and decoder were optimised to reproduce human GC biases; when applied to mouse inputs, the encoder maps them to out-of-distribution regions, and the decoder produces sequences that reflect human rather than mouse statistics. This failure is not simply a matter of k-mer composition (JSD = 0.355) but likely reflects deeper differences in promoter architecture, transcription factor binding site densities, and background nucleotide frequencies between the two species. Future work should explore multi-species training or domain-adaptation strategies to achieve cross-species regulatory sequence modelling

### Limitations and future directions

Three limitations warrant consideration. First, the current architecture operates on fixed-length sequences (363 bp). Although alignment analyses demonstrate effective handling through contextual extension or core extraction, native variable-length generation remains an open challenge for sequences whose functional boundaries are inherently heterogeneous.

Second, while the model captures *cis*-regulatory syntax, it does not incorporate higher-order chromatin organisation (e.g., topologically associating domains), which Sefer [39] and Sefer [40] have shown to be essential determinants of transcriptional competence in three-dimensional nuclear space.

Third, cross-species validation on *Mus musculus* sequences confirmed that the MT-AAE is a species-specific model. Applying the human-trained model to mouse mammary RNA-seq data yielded a TRTS accuracy of 0.051 and a GC MAE of 0.349—approximately 35 times the human in-domain error—reflecting the well-documented divergence between human and murine non-coding regulatory elements. This specificity is expected and appropriate for human-focused applications. Cross-species generalisation would require fine-tuning on target-species data or a multi-species training corpus with species-conditioning; extending MT-AAE to species-conditioned generation represents a natural direction for future work.

## Electronic supplementary material

Below is the link to the electronic supplementary material.

Supplementary Material 1

## Data Availability

The datasets used in this study were originally obtained from the NCBI Gene Database and are publicly available via Kaggle, as described in [58].

## Code availability

To ensure full reproducibility, the complete codebase, trained model checkpoints, hyperparameter configurations, and evaluation scripts are publicly available at https://github.com/Shamsu3100/mt-aae-genomics. The repository adheres to FAIR principles and provides all resources required to reproduce the experiments and results reported in this study.
