# Supplementary material for: Multi-task adversarial autoencoder for functional genomic element generation with preserved biophysical properties
Source: BioData Min. 2026 Aug 3;19:83. doi: 10.1186/s13040-026-00591-9 (PMC13726178; doi:10.1186/s13040-026-00591-9)
Supplement: Supplementary file 1 [file 13040_2026_591_MOESM1_ESM.pdf]

Supplementary Material S1: Detailed Mathematical Derivations

This section provides complete mathematical derivations for equations referenced in the main methodology, included here for reproducibility and technical completeness.

Encoder LSTM Dynamics

For each position $t \in [1, L]$, the bidirectional LSTM encoder computed forward and backward hidden states:

$$\overrightarrow{h_t} = \text{LSTM}^{\rightarrow}(\phi(s_t), \overrightarrow{h_{t-1}}; \theta^{\rightarrow}) \quad (1)$$

$$\overleftarrow{h_t} = \text{LSTM}^{\leftarrow}(\phi(s_t), \overleftarrow{h_{t-1}}; \theta^{\leftarrow}) \quad (2)$$

$$h_t = [\overrightarrow{h_t}; \overleftarrow{h_t}] \in \mathbb{R}^{512} \quad (3)$$

Type Embedding Transformation

Gene type information $y_{\text{type}} \in \{0, 1\}^C$ was embedded through fully connected layers stabilized by BatchNorm and Dropout:

$$y_{\text{embed}} = \text{BatchNorm}(\text{GeLU}(W_2 \cdot \text{GeLU}(W_1 y_{\text{type}} + b_1) + b_2)) \quad (4)$$

where $W_1 \in \mathbb{R}^{256\times C}$, $W_2 \in \mathbb{R}^{128\times 256}$.

Multi-Head Attention Mechanism

The attention mechanism enabling motif discovery was formalized as:

$$\text{Attention}(Q, K, V) = \text{softmax}\left(\frac{QK^T}{\sqrt{d_k}}\right)V \quad (5)$$

$$\text{MultiHead}(Q, K, V) = \text{Concat}(\text{head}_1, \ldots, \text{head}_h)W^O \quad (6)$$

$$\text{where head}_i = \text{Attention}(QW_i^Q, KW_i^K, VW_i^V)$$

The architecture employed $h = 8$ attention heads with key dimension $d_k = 32$.

Decoder LSTM Formulation

The decoder generated sequences through bidirectional LSTM dynamics:

$$h_t^d = \text{BiLSTM}([z_t; y_{\text{embed},t}], h_{t-1}^d; \theta_d)$$

Classification Head Architecture

The classification pathway processed pooled representations through deep layers:

$$h_{\text{pooled}} = \frac{1}{L}\sum_{t=1}^{L} h_t^d \quad (7)$$

$$h_1 = \text{BatchNorm}(\text{GeLU}(W_1 h_{\text{pooled}} + b_1)) \quad (8)$$

$$h_2 = \text{Dropout}(\text{BatchNorm}(\text{GeLU}(W_2 h_1 + b_2)), p = 0.3) \quad (9)$$

$$h_3 = \text{BatchNorm}(\text{GeLU}(W_3 h_2 + b_3)) \quad (10)$$

$$\hat{y}_{\text{class}} = \text{softmax}(W_c h_3 + b_c) \quad (11)$$

GC Regression Network

The GC content regression head predicted biophysical properties:

$$\hat{y}_{\text{GC}} = \sigma(W_{\text{GC}} \cdot \text{GeLU}(W_{\text{GC1}} h_{\text{pooled}} + b_{\text{GC1}}) + b_{\text{GC}})$$

Efficiency Measurement Formulas

Computational efficiency was quantified using:

$$\text{Total Training Time} = T_{\text{end}} - T_{\text{start}} \tag{12}$$

$$\text{Time per Epoch} = \frac{T_{\text{total}}}{E_{\text{total}}} \tag{13}$$

$$\text{Sequence Generation Speed} = \frac{N_{\text{gen}}}{T_{\text{gen}}} \tag{14}$$

$$\text{End-to-End Pipeline Efficiency} = \frac{N_{\text{gen}}}{T_{\text{pipeline}}} \tag{15}$$

Statistical Validation Metrics

Distributional similarity between synthetic and natural sequences was quantified using:

$$\text{JSD}(P \parallel Q) = \frac{1}{2} D_{\text{KL}}(P \parallel M) + \frac{1}{2} D_{\text{KL}}(Q \parallel M), \quad M = \frac{1}{2}(P + Q) \tag{16}$$

$$D_{n,m} = \sup_x |F_{1,n}(x) - F_{2,m}(x)| \tag{17}$$

$$d = \frac{\mu_1 - \mu_2}{\sqrt{\frac{(n_1-1)\sigma_1^2 + (n_2-1)\sigma_2^2}{n_1+n_2-2}}} \tag{18}$$

$$\text{CV}_{\text{TRTS}} = \frac{1}{3} \sum_{k=1}^{3} \text{TRTS}_k, \quad \sigma_{\text{TRTS}} = \sqrt{\frac{1}{2} \sum_{k=1}^{3} (\text{TRTS}_k - \text{CV}_{\text{TRTS}})^2} \tag{19}$$

Sequence Alignment Metrics

Local sequence alignment analysis used BLASTN with metrics:

$$\text{Identity} = \frac{\text{Matches}}{\text{Aligned Positions}}, \quad \text{Coverage} = \frac{\text{Aligned Length}}{\text{Original Length}}$$

S2: Per-Class Metrics for Foundation Model TRTS Evaluation

Table 1 reports the precision, recall, F1-score, and support for every class in the real-sequence test set for the three foundation-model evaluators described in Section **??**. The macro-averaged F1 and weighted accuracy correspond to the values given in the main text (Table **??**).

Table 1: Per-class TRTS results for the frozen-encoder evaluators. ”Accuracy” corresponds to the weighted-average recall across all classes; ”Macro F1” represents the unweighted mean of per-class F1-scores.

(a) DNABERT-2 (117M, CLS pooling)

Class	Precision	Recall	F1-Score	Support
BIOLOGICAL_REGION	0.6364	0.1167	0.1972	60
PROTEIN_CODING	0.4000	0.1754	0.2439	57
PSEUDO	0.5490	0.3836	0.4516	73
enhancer	0.5385	0.2188	0.3111	32
intron	0.3421	0.8298	0.4845	47
ncRNA	0.5526	0.6000	0.5753	35
promoter	0.5161	0.4848	0.5000	33
rRNA	0.4857	0.6800	0.5667	25
snoRNA	0.2593	0.5385	0.3500	13
tRNA	0.0811	0.4286	0.1364	7
**Macro Avg**	0.4361	0.4456	**0.3817**	382
**Weighted Avg**	0.4891	0.4058	0.3867	382

(b) DNABERT-S (21M, CLS pooling)

Class	Precision	Recall	F1-Score	Support
BIOLOGICAL_REGION	0.6207	0.3000	0.4045	60
PROTEIN_CODING	0.3571	0.1754	0.2353	57
PSEUDO	0.5686	0.3973	0.4677	73
enhancer	0.4667	0.2188	0.2979	32
intron	0.3486	0.8085	0.4872	47
ncRNA	0.7059	0.3429	0.4615	35
promoter	0.4565	0.6364	0.5316	33
rRNA	0.4889	0.8800	0.6286	25
snoRNA	0.2667	0.3077	0.2857	13
tRNA	0.1111	0.4286	0.1765	7
**Macro Avg**	0.4391	0.4495	**0.3977**	382
**Weighted Avg**	0.4887	0.4293	0.4152	382

(c) HyenaDNA (tiny-1k, last-token pooling)

Class	Precision	Recall	F1-Score	Support
BIOLOGICAL_REGION	0.2941	0.1667	0.2128	60
PROTEIN_CODING	0.2857	0.1404	0.1882	57
PSEUDO	0.1765	0.0411	0.0667	73
enhancer	0.1136	0.1562	0.1316	32
intron	0.1000	0.1064	0.1031	47
ncRNA	0.0857	0.0857	0.0857	35
promoter	0.0667	0.0909	0.0769	33
rRNA	0.0263	0.0400	0.0317	25
snoRNA	0.0328	0.1538	0.0541	13
tRNA	0.0333	0.1429	0.0541	7
**Macro Avg**	0.1215	0.1124	**0.1005**	382
**Weighted Avg**	0.1614	0.1073	0.1174	382

S3: De Novo Motif Discovery and Structural Fidelity Verification of Generated Promoters

References

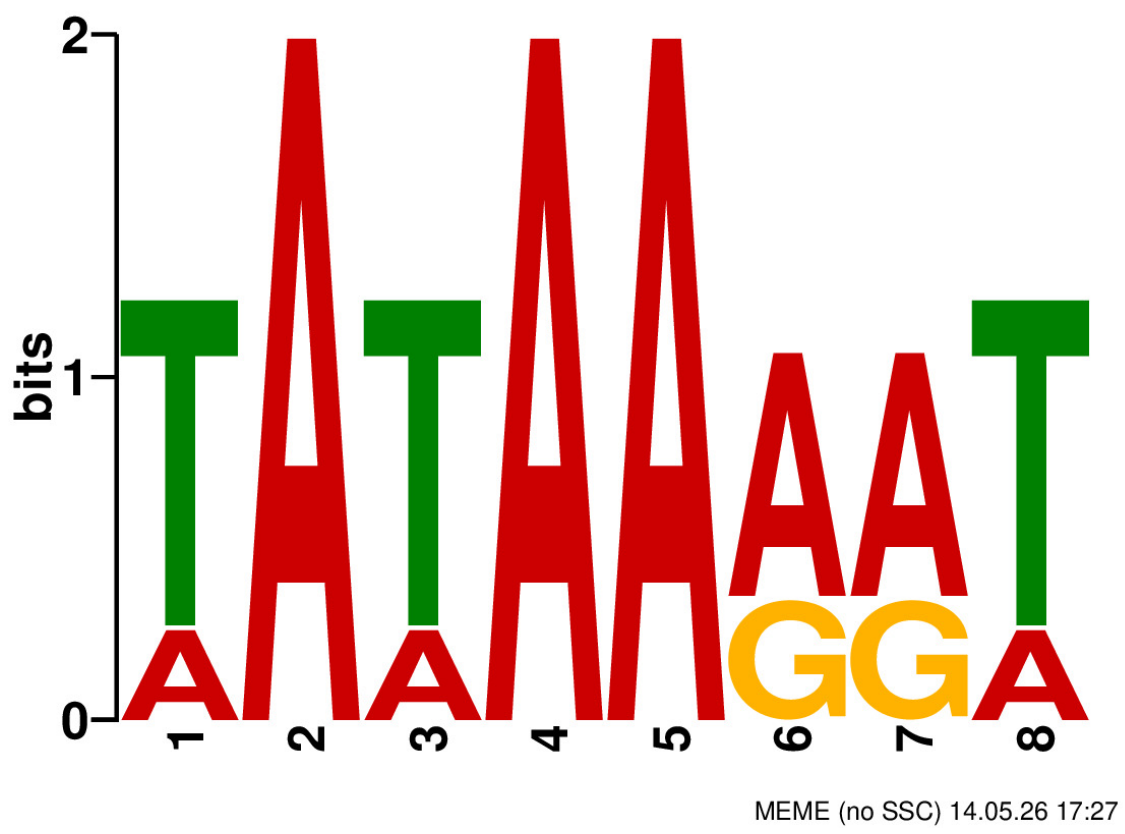

(a) Discovered TATA-box motif logo (E-value = 3.2×10^{-30}).

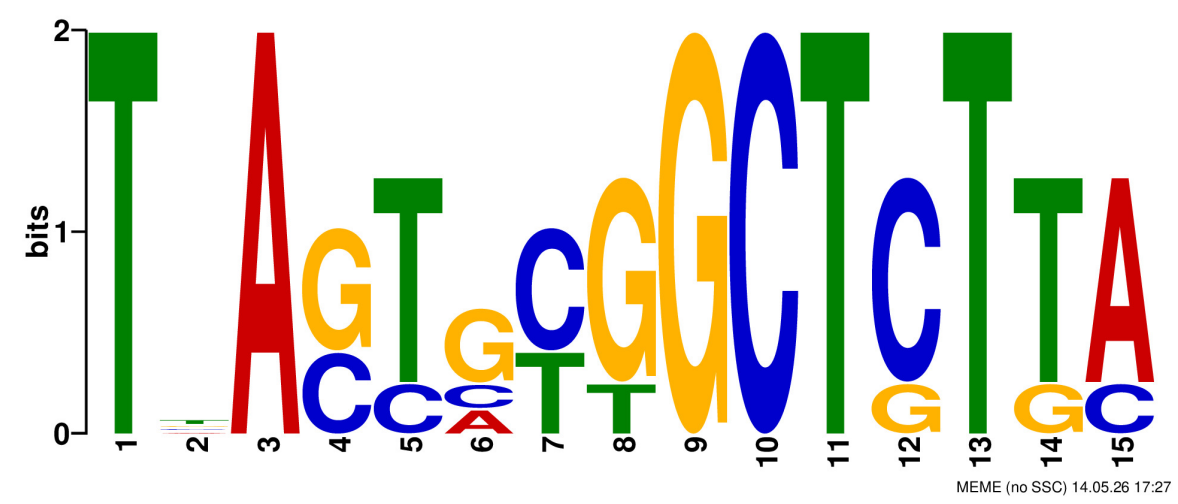

(b) Secondary structural sequence motif logo discovered via MEME.

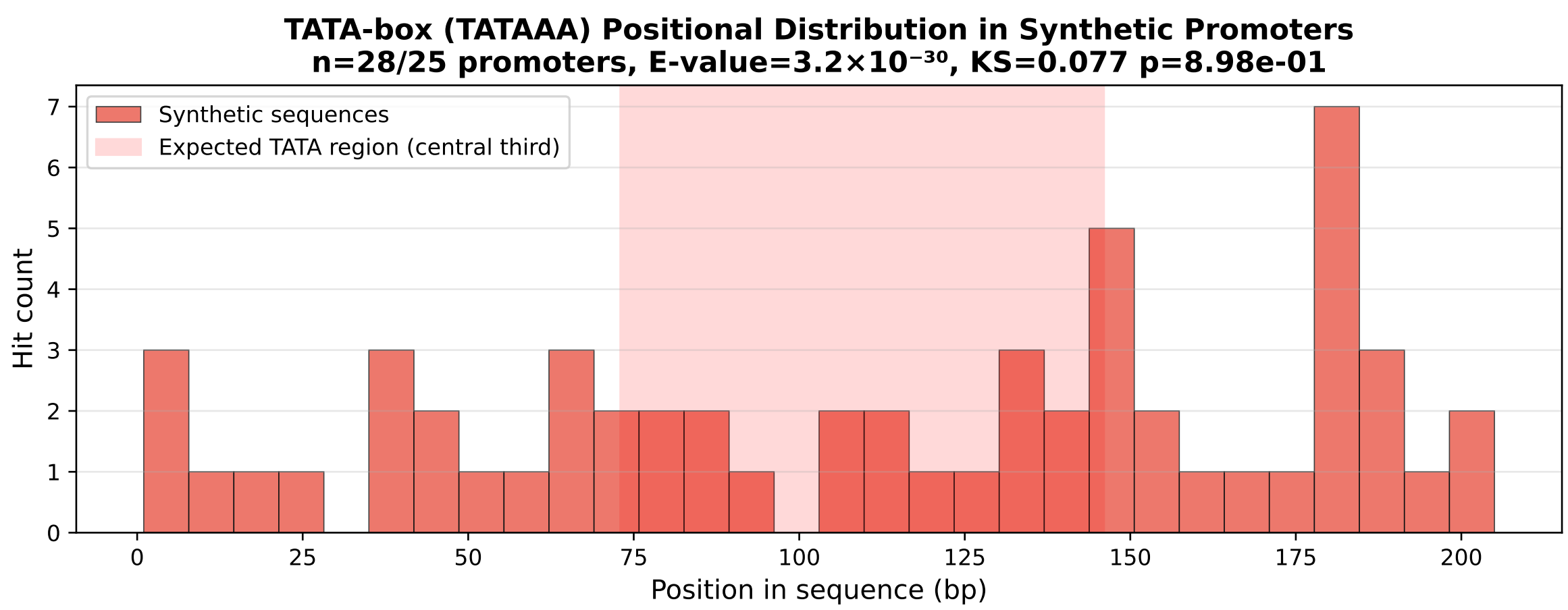

(c) TATA-box (`TATAAA`) spatial distribution profile across synthetic promoter sequences ($n = 25$). The shaded area defines the targeted central third region (75–142 bp).

Figure 1: Supplementary structural verification of synthetic sequences. (a–b) Sequence logos illustrating the information content (bits) and nucleotide conservation across discovered motifs. (c) Empirical coordinate distribution of the generated TATA-box sequence components relative to expected biological localization landmarks.
